# MicroRNA Signatures in Endometrial Receptivity—Unlocking Their Role in Embryo Implantation and IVF Success: A Systematic Review

**DOI:** 10.3390/biomedicines13051189

**Published:** 2025-05-13

**Authors:** Charalampos Voros, Antonia Varthaliti, Diamantis Athanasiou, Despoina Mavrogianni, Kyriakos Bananis, Antonia Athanasiou, Aikaterini Athanasiou, Anthi-Maria Papahliou, Constantinos G. Zografos, Panagiota Kondili, Maria Anastasia Daskalaki, Dimitris Mazis Kourakos, Dimitrios Vaitsis, Marianna Theodora, Panagiotis Antsaklis, Dimitrios Loutradis, Georgios Daskalakis

**Affiliations:** 1 1st Department of Obstetrics and Gynecology, ‘Alexandra’ General Hospital, National and Kapodistrian University of Athens, 80 Vasilissis Sofias Avenue, 11528 Athens, Greece; antonia.varthaliti@hotmail.com (A.V.); depy.mavrogianni@yahoo.com (D.M.); anthipapahliou@gmail.com (A.-M.P.); giotakondyli27@gmail.com (P.K.); md181341@students.euc.ac.cy (M.A.D.); martheodr@gmail.com (M.T.); panosant@gmail.com (P.A.); gdaskalakis@yahoo.com (G.D.); 2 IVF Athens Reproduction Center V. Athanasiou, 15123 Maroussi, Greece; diamathan16@gmail.com (D.A.); antoathan16@gmail.com (A.A.); aikathan16@gmail.com (A.A.); 3 King’s College Hospitals NHS Foundation Trust, London SE5 9RS, UK; kyriakos.bananis@nhs.net; 4 2nd Surgical Department, General Hospital of Athens “LAIKO”, 11527 Athens, Greece; koszogra92@hotmail.com; 5 Rea Maternity Hospital S.A., Avenue Siggrou 383 & Pentelis 17, P. Faliro, 17564 Athens, Greece; mazisdimitris@gmail.com; 6 Athens Medical School, National and Kapodistrian University of Athens, 15772 Athens, Greeceloutradi@otenet.gr (D.L.); 7 Fertility Institute—Assisted Reproduction Unit, Paster 15, 11528 Athens, Greece

**Keywords:** endometrial receptivity, microRNAs, implantation failure, assisted reproductive technology (ART), embryo implantation, lncRNAs, circular RNAs (circRNAs), HOXA10, decidualization, molecular biomarkers

## Abstract

**Background:** Endometrial receptivity is crucial for successful embryo implantation in assisted reproductive technologies (ARTs). MicroRNAs (miRNAs), long non-coding RNAs (lncRNAs), and circular RNAs (circRNAs) have emerged as important post-transcriptional regulators of endometrial function, although their diagnostic and molecular functions are poorly understood. **Methods:** A systematic review was conducted following PRISMA 2020 principles and registered in PROSPERO (CRD420251001811). We looked at 28 peer-reviewed publications published between 2010 and 2025 that used endometrial tissue, blood, uterine fluid, saliva, and embryo culture medium to study miRNAs and other non-coding RNAs in endometrial receptivity, recurrent implantation failure (RIF), and infertility. **Results:** MiRNAs like miR-145, miR-30d, miR-223-3p, and miR-125b influence implantation-related pathways such as HOXA10, LIF-STAT3, PI3K-Akt, and Wnt/β-catenin. Dysregulated expression profiles were linked to inadequate decidualization, immunological imbalance, and poor angiogenesis. CeRNA networks that include lncRNAs (e.g., H19 and NEAT1) and circRNAs (e.g., circ_0038383) further regulate miRNA activity. Non-invasive biomarkers derived from plasma, uterine fluid, and embryo media showed high prediction accuracy for implantation outcomes. **Conclusions:** MiRNA signatures offer a functional and diagnostic blueprint for endometrial receptivity. This systematic review provides a timely and thorough synthesis of the existing literature, with the goal of bridging the gap between molecular discoveries and therapeutic applications. By emphasizing both the mechanistic importance and diagnostic value of certain miRNA signatures, it paves the way for future precision-based techniques in embryo transfer and endometrial assessment in ART.

## 1. Introduction

Embryo implantation is a critical stage in human reproduction, facilitated by a delicate interplay between the embryo and the endometrium [1]. Despite technological breakthroughs in assisted reproductive technologies (ARTs), the implantation rate per embryo transfer is still frustratingly low, averaging around 30–40% even under ideal settings [2]. One of the key causes of this inefficiency is the failure of endometrial receptivity—a temporary functional circumstance in which the endometrium develops the ability to absorb and nourish the implanting blastocyst [3]. This condition is limited to the window of implantation (WOI), which normally lasts between days 19 and 23 of the menstrual cycle and is distinguished by a tightly regulated transcriptome, epigenomic, and proteomic landscape. In this setting, recurrent implantation failure (RIF)—commonly defined as the failure to produce a clinical pregnancy after transferring at least two-to-four good-quality embryos in several IVF cycles—continues to be a serious clinical challenge [4]. RIF affects up to 10% of IVF patients. Evidence suggests that endometrial dysregulation, rather than embryo abnormalities, is responsible for the bulk of these failures [5,6]. However, current approaches for measuring endometrial receptivity are mostly empirical and lacking in molecular accuracy, emphasizing the critical need for mechanism-based diagnostics and biomarkers.

### 1.1. MicroRNAs: Biogenesis, Function, and Mechanistic Roles

MicroRNAs (miRNAs) are small, non-coding RNA molecules that influence gene expression in almost all eukaryotic cells after transcription. They are approximately 21–25 nucleotides long. They serve critical functions in reproductive physiology, such as endometrial remodeling, immunological tolerance, angiogenesis, stromal cell differentiation, and embryo–maternal communication during the WOI. Aberrant miRNA expression profiles are highly related to RIF, endometriosis-associated infertility, and other reproductive diseases, making them appealing targets for both diagnostic and therapeutic intervention [5,6,7,8].

MiRNA biogenesis is a multistep process that begins in the nucleus and ends in the cytoplasm, where they exert gene-silencing effects. MiRNA genes are transcribed by RNA polymerase II or III, resulting in lengthy primary miRNA transcripts (pri-miRNAs) with stem–loop structures. The Drosha–DGCR8 microprocessor complex processes pri-miRNAs in the nucleus, resulting in around 70 nucleotide precursor miRNAs. These are then delivered to the cytoplasm by Exportin-5 in a Ran-GTP-dependent manner. Dicer, an RNase III enzyme, cleaves the pre-miRNA in the cytoplasm to form a ~22-nucleotide miRNA duplex. The guide strand of this duplex is integrated into the RNA-induced silencing complex (RISC), whereas the passenger strand is normally destroyed. The miRNA-RISC complex binds to complementary sequences, typically inside the 3′ untranslated region (3′-UTR) of target messenger RNAs (mRNAs), resulting in either translational suppression or mRNA destruction, depending on the degree of complementarity [6,7].

Mature miRNAs, once incorporated into RISC, serve as important post-transcriptional regulators of gene expression [9]. A single miRNA can target many mRNAs, and vice versa, creating a complex and dynamic regulatory network [10]. These interactions influence a variety of cellular activities in the endometrium, especially during the secretory phase, when the tissue becomes more susceptible to embryo implantation.

MiRNAs act as rheostats, fine-tuning gene expression in various essential biological processes related to endometrial receptivity, including the following:

- MiRNAs control the development of stromal fibroblasts into decidual cells, which produce prolactin and IGFBP1. For example, miR-21-5p, miR-193b-3p, and miR-17-5p have been linked to regulating endoplasmic reticulum stress (ERS) and the unfolded protein response (UPR) during decidualization. Disruption in these stress-adaptation mechanisms may cause poor implantation and inflammations [6].
- Immune modulation: The maternal immune system must accept the semi-allogeneic embryo. MiRNAs like miR-146a, miR-125b, and miR-124-3p impact the expression of cytokines, interleukins, and immunological checkpoint molecules, including leukemia inhibitory factor (LIF), IL-11, and SOCS1, influencing the Th1/Th2 cytokine balance, macrophage polarization, and T-regulatory cell recruitment [5,6].
- Angiogenesis and vascular remodeling: The endometrial bed must be extensively vascularized before embryo implantation can occur. RIF patients exhibit variable expression of miRNAs (miR-27a, miR-20a, and miR-126), which target angiogenic regulators such as VEGFA, HIF-1α, and FLT1 [11].
- Extracellular matrix (ECM) remodeling: Tissue invasion and ECM breakdown are required for proper implantation. MiR-29c, miR-145, and miR-30b regulate ECM components such as MMP26, TIMP3, and integrins (e.g., ITGβ3), which are commonly downregulated in women with RIF [5,6].
- miRNAs like miR-149 and miR-33a play a role in modulating the Wnt/β-catenin signaling pathway, which is crucial for implantation. These miRNAs control epithelial–mesenchymal transition (EMT), trophoblast invasion, and uterine receptivity by directly or indirectly targeting WNT3A and its downstream effectors [12].

In addition to differential expression, mutations in miRNA genes or their target sites can affect binding efficiency and regulatory impact, including the following:

- miR-146aC>G and miR-196a2T>C polymorphisms were shown to be strongly linked with increased RIF risk in Korean women, possibly because to differential maturation and expression of these miRNAs and their downstream inflammatory pathways [5].
- SNPs in miR-125a, miR-365b, and miR-149 have been linked to poor reproductive outcomes, including fetal growth limitation, due to inadequate regulation of Wnt/β-catenin signaling and angiogenesis pathways [12].

Furthermore, the interaction between miRNAs and epigenetic changes, such as DNA methylation of miRNA promoters or chromatin remodeling, adds a new regulatory layer that modulates endometrial receptivity. Some studies imply that hypermethylation of miRNA genes may suppress their production in diseased endometrium, although this is an area that requires further exploration.

MiRNAs function within larger competing endogenous RNA (ceRNA) networks, in which long non-coding RNAs (lncRNAs) and circular RNAs (circRNAs) sequester individual miRNAs, adjusting their bioavailability and mitigating their effects. These networks increase the intricacy and plasticity of post-transcriptional regulation.

- For example, circ_0038383 was discovered to sponge miR-196b-5p, thereby upregulating HOXA9, a critical transcription factor for stromal cell development and embryo–maternal communications [6].
- LncRNAs H19 and NEAT1, which are abundant in mid-secretory endometrium, influence miR-29c, miR-20a, and other miRNAs involved in decidualization and immunological tolerance [13].

### 1.2. Molecular Pathways Governed by miRNAs in Endometrial Receptivity

The transformation of the human endometrium to a receptive condition is a meticulously planned process that involves a series of hormonal, cellular, and molecular interactions [14]. These include transforming uterine stromal fibroblasts into decidual cells, modulating immune cell populations, altering the extracellular matrix (ECM), angiogenesis, and regulating cell adhesion and intercellular signaling [15]. MicroRNAs (miRNAs) regulate gene expression post-transcriptionally, which is crucial to this multilayered biological program [16]. These tiny non-coding RNAs function as potent molecular switches, repressing, fine-tuning, or buffering gene expression in response to endocrine signals, metabolic states, and environmental stimuli. Endometrial biopsies, in vitro experiments, and transcriptome analysis all provide evidence that miRNAs play key roles in regulating the biological pathways required for endometrial receptivity and embryo implantation [17].

The homeobox (HOX) gene family, particularly HOXA10 and HOXA11, which are master transcriptional regulators of uterine development and endometrial function, is one of the basic pathways in the endometrium that miRNAs affect [18]. These genes regulate the production of downstream targets like integrins, IGFBPs, and cytokines, which are required for stromal cell differentiation and embryo adhesion. Upregulation of particular miRNAs, notably miR-135a and miR-135b, has been found to directly inhibit HOXA10 expression in women with recurrent implantation failure (RIF) or endometriosis-associated infertility [13]. Suppression causes downstream dysfunctions, such as decreased production of integrin β3 (ITGB3), a critical cell adhesion molecule; and LIF, a mediator of embryo–endometrium communication. Other miRNAs, like as miR-27a-3p, target HOXA10 and are more prevalent in infertile women, emphasizing the sensitivity of this developmental pathway to miRNA-mediated regulation [19].

The LIF-STAT3 signaling pathway is closely linked to the HOX regulatory axis, and it plays an important role throughout the implantation window by modulating immunological tolerance, epithelial receptivity, and stromal support for the implanting blastocyst [20]. Dysregulation of this mechanism is frequent in women with RIF. MiR-30d-5p, which normally increases LIF expression, is dramatically reduced in non-receptive endometrial settings. This downregulation causes the upregulation of Suppressor of Cytokine Signaling 1 (SOCS1), a negative regulator of STAT3 phosphorylation and activity. The end effect is a decreased STAT3 response and reduced LIF signaling, which impairs endometrial receptivity. In addition, miR-125b and miR-124-3p have been demonstrated to directly inhibit LIF mRNA, and both are higher in women with endometriosis and unexplained infertility [21]. The cumulative effect of various miRNAs on LIF-STAT3 signaling demonstrates how endometrial receptivity can be harmed at numerous levels within a single important pathway.

MiRNAs also affect the Wnt/β-catenin signaling system, which governs EMT, cell polarity, decidualization, and trophoblast invasion. Wnt ligands, such as WNT3A, are required for endometrial remodeling and are highly regulated throughout the mid-secretory phase [22].

MiRNAs like as miR-149, miR-33a, and miR-125a have been demonstrated to directly or indirectly target WNT3A, and genetic variations in these miRNA genes, or their binding sites can further influence Wnt signaling efficiency [23]. Disruption of Wnt/β-catenin activity causes poorer decidualization, aberrant expression of adhesion molecules, and failure to create an implantation-competent endometrium. Genetic variations in miR-33a and WNT3A have been postulated as potential prenatal indicators in women with fetal growth restriction (FGR), demonstrating the pathway’s broader relevance beyond implantation alone [24].

Regulation of extracellular matrix (ECM) remodeling and cell adhesion is also critical in the development of a responsive endometrium. The constant remodeling of ECM components enables trophoblast invasion and stable embryo attachment [25]. Several miRNAs regulate the enzymes that cause these structural alterations. For example, miR-29c and miR-145 target matrix metalloproteinases (MMPs), such as MMP26, as well as tissue inhibitors of metalloproteinases (TIMPs), regulating ECM protein breakdown [26]. RIF patients had altered miR-29c expression, resulting in unbalanced MMP/TIMP activity and a stiff, non-receptive endometrial interface. MiRNAs regulate integrins, including ITGβ3 [27]. Their expression is reduced by miRNAs such as miR-125b, which contributes to poor cell adhesion at the maternal–fetal interface.

The endometrial immune milieu must shift from pro-inflammatory to tolerogenic during the implantation window. This is somewhat facilitated by miRNA-regulated cytokine networks. MiR-146a, miR-155, and miR-21 have been found to regulate the balance of Th1 and Th2 immune responses by affecting the activity of uterine natural killer (uNK) cells, dendritic cells, and T-regulatory cells [28]. Women with RIF have abnormal miRNA expression, which increases pro-inflammatory mediators like IL-1β and TNF-α while decreasing anti-inflammatory cytokines like IL-10 and TGF-β [29]. MiRNAs like miR-17-5p, miR-21-5p, and miR-193b-3p cross-regulate immune signaling and ER stress pathways, leading to defective decidualization and implantation failure. These miRNAs modulate key unfolded protein response (UPR) effectors like PERK, IRE1α, and CHOP [6].

Finally, miRNAs influence hormonal signaling cascades required for the endometrium’s transition from proliferative to secretory phase. MiRNA interference has a significant impact on estrogen and progesterone receptor levels, as well as downstream effectors such as IGFBP1. For example, miR-196a2 and miR-222 have been demonstrated to inhibit ESR1 (estrogen receptor alpha), hence affecting estrogen-mediated proliferation. Meanwhile, abnormal expressions of miR-29a, miR-194, and miR-135b affect progesterone responsiveness and stromal differentiation. These hormonal effects are exacerbated further by cyclic change in miRNA expression throughout the menstrual cycle, underlining the importance of temporal specificity when assessing endometrial biomarkers.

### 1.3. Non-Coding RNA Crosstalk and ceRNA Networks in Endometrial Receptivity and Implant Failure

Beyond their specific tasks, microRNAs (miRNAs) are now known to function within a larger regulatory framework that includes several RNA-RNA interactions. These interactions take place among several types of non-coding RNAs, including long non-coding RNAs (lncRNAs), circular RNAs (circRNAs), and messenger RNAs (mRNAs), constituting what is known as the competitive endogenous RNA (ceRNA) network [30]. This network functions as a sophisticated buffering system, fine-tuning gene expression and protein production by competitive miRNA binding to various RNA species with shared miRNA response elements (MREs) [31]. This molecular interaction in the endometrium is an important layer of post-transcriptional control that regulates cellular differentiation, immunological signaling, and receptivity-related gene expression throughout the implantation window [32]. Dysregulation of ceRNA networks is being increasingly linked to the molecular pathophysiology of RIF and associated reproductive diseases.

The ceRNA hypothesis posits that RNAs with similar MREs might successfully “compete” for miRNA binding, hence varying the availability of active miRNAs. In this system, a decrease in one ceRNA species, such as a specific lncRNA, may result in enhanced repression of its target mRNAs due to an abundance of free miRNA [33]. In contrast, increasing the abundance of a sponge-like circRNA or lncRNA can adsorb miRNAs and reduce mRNA target inhibition. This balancing act is especially important in hormone-responsive tissues such as the endometrium, where modest changes in miRNA activity can have a significant impact on gene networks that drive decidualization, immunological tolerance, and epithelial remodeling [34]. Recent transcriptomic and bioinformatics investigations on secretory-phase endometria in women with RIF have identified altered ceRNA networks comprising both circular and long non-coding RNAs. One notable example is the circ_0038383/miR-196b-5p/HOXA9 axis, in which circ_0038383 acts as a sponge for miR-196b-5p, controlling the expression of HOXA9, a transcription factor required for stromal differentiation and epithelial receptivity. In RIF patients, downregulation of circ_0038383 increases the availability of miR-196b-5p, which reduces HOXA9 expression, thus impairing decidualization and disrupting endometrial-embryonic communication [35]. This route demonstrates how circular RNAs can indirectly regulate critical implantation genes via miRNA sequestration, leading to molecular abnormalities in the receptive endometrium.

Similarly, long non-coding RNAs have been found to be key endometrial gene regulators. LncRNA H19, one of the most widely investigated lncRNAs in reproductive organs, is known to affect miRNAs like miR-29c and miR-200 family members [36]. In the context of implantation, H19 expression rises during the mid-secretory phase and helps to stromal cell decidualization by sponging miRNAs that would otherwise suppress IGF2 and integrin genes.

Reduced H19 expression in women with implantation failure has been associated with increased miRNA activity and downregulation of genes involved in ECM remodeling and cell adhesion [37]. NEAT1, an lncRNA, regulates TGF-β signaling and interacts with miR-146b to modulate endometrial immunological function [38]. Disruption of these lncRNA-centered ceRNA networks causes inappropriate inflammatory signaling, excessive ER stress responses, and poor immunological tolerance, all of which are associated with RIF.

Furthermore, the dynamic nature of the endometrial transcriptome during the menstrual cycle complicates ceRNA regulation. Hormonal signals, namely progesterone and estrogen, influence the expression of both miRNAs and their lncRNA or circRNA rivals [39]. During the receptive phase, particular ceRNA interactions promote anti-inflammatory and pro-differentiation gene expression. Hormonal imbalances or altered miRNA turnover might undermine the ceRNA network’s buffering ability, resulting in overactivation or silence of critical pathways such LIF-STAT3, Wnt/β-catenin, and PI3K-Akt [40]. The importance of ceRNA dysregulation in reproductive diseases is reinforced by research tying these networks to endometriosis and adenomyosis, both of which are known to impair implantation potential. In these illnesses, abnormal expression of particular circRNAs and lncRNAs changes the competitive landscape for miRNA binding, shifting the balance away from receptivity-supporting gene expression. Endometrial samples from adenomyosis patients, for example, show alterations in ceRNA interactions involving miR-21-5p, which has been linked to defective ECM remodeling and enhanced fibrotic signaling [41].

In terms of molecular pathophysiology, alterations in ceRNA networks indicate a loss of regulatory resilience in the endometrial environment. Unlike single-gene mutations, these disruptions are frequently caused by small quantitative variations in RNA quantity or miRNA-lncRNA ratios. However, the repercussions can be systemic, resulting in the coordinated misregulation of several target genes involved in implantation. CeRNA networks are particularly intriguing for biomarker development because they can reflect system-level dysregulation through detectable changes in non-coding RNA levels in tissue or biofluids.

### 1.4. miRNAs as Biomarkers

MicroRNAs (miRNAs)’ distinct molecular properties—their small size, stability in bodily fluids, and dynamic expression profiles—make them very intriguing candidates for non-invasive diagnostics in reproductive medicine [42]. In the context of endometrial receptivity and RIF, miRNAs’ diagnostic utility stems directly from their functional integration into critical pathways governing stromal differentiation, immune modulation, hormonal responsiveness, and embryo–endometrial crosstalk. MiRNAs regulate these processes at the post-transcriptional level, so their expression patterns reflect the endometrium’s functional state during the window of implantation (WOI), providing a molecular readout that can be used for patient stratification, cycle optimization, and embryo transfer-timing personalization [43].

One of the most appealing aspects of miRNAs as biomarkers is their detectability in a variety of bodily fluids, including blood plasma, serum, uterine fluid, saliva, and even wasted embryo culture material [44]. This widespread extracellular dispersion is assisted by their packaging into extracellular vesicles (EVs), such as exosomes, or by binding to RNA-binding proteins (e.g., Argonaute-2) or lipoproteins (e.g., HDL), which shield them from enzymatic breakdown [45].

These characteristics make miRNAs extremely stable, even under inferior sample processing settings, and appropriate for liquid biopsy-based diagnostics. Unlike standard endometrial biopsy, which is invasive and disrupts the cycle, liquid biopsy techniques can give real-time, non-invasive assessments of endometrial receptivity, allowing for repeated sample and longitudinal monitoring.

Clinical trials have begun to confirm the diagnostic value of circulating and tissue-specific miRNA signatures. For example, Chen et al. (2023) [43] created a plasma-based miRNA classifier using the PanelChip^®^ technology that achieved over 95% diagnostic accuracy in discriminating between receptive and non-receptive endometrial profiles. The model included miRNAs such as miR-30b, miR-181, and miR-223-3p, which are all mechanistically linked to networks involved in hormone sensitivity and immunological control. A recent study by the same group, Chen et al. (2024) [46], expanded on this strategy by developing the MIRA test, which combines blood-based miRNA profiling with artificial intelligence-driven cycle analysis to personalize progesterone support and embryo transfer time. In clinical validation, the MIRA test rectified the issue in more than 90% of patients with previously unsuccessful implantation, resulting in significantly better pregnancy outcomes.

Beyond blood, uterine fluid has emerged as a particularly useful source of miRNA biomarkers because it comes into direct contact with the luminal epithelium during embryo apposition and adhesion. Von Grothusen et al. (2022) [27] found that particular miRNAs—such as miR-486-5p, miR-92a-3p, and miR-99b-5p—are more abundant in uterine fluid samples from RIF patients than in fertile controls. These miRNAs are important in cellular adhesion, ECM remodeling, and cytokine signaling, and their dysregulation corresponds to functional abnormalities seen histologically and transcriptomically in the non-receptive endometrium. Importantly, these findings were confirmed in endometrial biopsies from the same people, bolstering the argument for uterine-fluid miRNAs as accurate biomarkers of local tissue activity.

In a more recent development, miRNAs extracted from embryo wasted culture medium have demonstrated potential for non-invasively monitoring embryo viability and predicting implantation results. Omes et al. (2023) [47] evaluated culture medium from developing embryos and discovered a collection of extracellular miRNAs, including miR-21-5p and miR-661, that were more abundant in embryos that successfully reached the blastocyst stage. These miRNA target genes control cell cycle progression, oxidative stress response, and metabolic regulation, all of which are essential for early embryonic development. Although still in its early stages, this strategy opens up the exciting option of measuring endometrial receptivity and embryo competency at the same time, using integrated miRNA profiling techniques.

Saliva has also emerged as an unorthodox but easily accessible diagnostic fluid for miRNA analysis. Bendifallah et al. (2024) [8] conducted a prospective study to assess the capacity of salivary miRNA profiles to detect superficial peritoneal endometriosis (SPE), which is frequently associated with unexplained infertility and low implantation rates. Using next-generation sequencing and machine learning-based classification, the authors discovered a set of 89 miRNAs that accurately separated SPE from other endometriosis phenotypes with 100% sensitivity and specificity. Given that SPE is often undetected by imaging and frequently requires diagnostic laparoscopy, our non-invasive technique provides a transformative diagnostic option that may also inform implantation risk.

MiRNAs have a higher diagnostic value due to their tissue specificity and cycle-dependent expression. Several studies have found that miRNA expression in the endometrium changes throughout the menstrual cycle, exhibiting unique profiles in the proliferative, early secretory, and mid-secretory phases. MiRNAs such as miR-30d, miR-135b, miR-145, and miR-125b exhibit significant variations during the WOI, indicating their regulatory involvement in decidualization, immunological quiescence, and stromal reprogramming [48]. These temporal dynamics suggest that miRNAs can act not just as static diagnostic markers but also as functional surrogates of endometrial status, delivering real-time information on tissue readiness for implantation.

Despite the potential, clinical adoption of miRNA-based diagnostics comes with numerous hurdles. Standardization of sample collection, RNA extraction techniques, normalization strategies, and data interpretation is still a significant obstacle to reproducibility and clinical use. Furthermore, many existing studies are based on small cohorts, lack external validation, and frequently use diverse platforms (e.g., quantitative real-time polymerase chain reaction (RT-PCR), microarrays, and NGS), making cross-study comparisons challenging. Addressing these problems would necessitate collaboration among clinical and research institutes, as well as integration with multi-omics platforms and machine-learning algorithms to improve accuracy and generalizability.

### 1.5. Current Limitations and Future Directions

While an increasing body of evidence highlights the mechanistic and diagnostic importance of microRNAs (miRNAs) and other non-coding RNAs in endometrial receptivity and implantation, many significant limitations now prevent their seamless translation from lab to bedside [17]. These constraints apply to biological interpretation, methodological standardization, clinical implementation, and translational reproducibility, all of which must be addressed before miRNA-based diagnostics or therapies can be implemented in ordinary reproductive practice.

One significant constraint is the biological complexity and tissue-specific context of miRNA function. Individual miRNAs have been shown to influence major endometrial receptivity regulators, including HOXA10, LIF, IGFBP1, MMP26, and STAT3; however, these molecules rarely function independently. They reside inside overlapping, redundant, and frequently compensating gene regulatory networks. As a result, the effect of any one miRNA on endometrial physiology may be limited and context-dependent, impacted by hormonal state, cellular heterogeneity, and temporal variations throughout the menstrual cycle. The endometrial transcriptome’s dynamic and cyclic nature provides significant biological variability, confounding the interpretation of static miRNA data collected at a single timepoint. To effectively quantify cycle-dependent miRNA expression and functional alterations, future research must include longitudinal sampling, ideally timed to coincide with the luteal phase.

Another continuing difficulty is a lack of methodological consistency across miRNA research. Variations in sample types (e.g., plasma, uterine fluid, and tissue biopsies), collection processes, RNA extraction techniques, and normalization strategies have posed substantial challenges to reproducibility [49]. For example, some studies use endogenous small RNAs (e.g., U6 or SNORD48) to normalize, whereas others use exogenous spike-in controls or global mean normalization. These methodological differences provide contradictory results, hampering meta-analytic efforts and cross-study comparisons [50]. Standardization of miRNA quantification procedures, data reporting, and quality control standards is required to build a strong foundation for biomarker development and validation.

Clinical translation is further hampered by small sample numbers and the absence of external validation in many published investigations. Several miRNA classifiers have exhibited excellent diagnostic accuracy in pilot populations, but few have been independently replicated in larger, multicenter studies. Furthermore, studies frequently lack stratification by crucial clinical characteristics such as age, BMI, ovarian reserve, presence of endometriosis or adenomyosis, and past ART results, all of which might influence miRNA levels. A more rigorous, stratified study design is required to evaluate the specificity and sensitivity of miRNA signatures in different subpopulations of infertility, as well as to separate disease-specific signals from broader physiological variation.

In terms of diagnostic integration, the clinical value and cost-effectiveness of miRNA-based assays need to be proved. It is unclear if miRNA tests will outperform or supplement current methods, such as histology dating, ERA testing, and transvaginal ultrasound-based evaluations. Furthermore, legal and logistical barriers—such as assay repeatability, CLIA validation, and insurance coverage—must be overcome before such diagnostics may be widely utilized. An important future path will be the creation of multi-analyte panels that combine miRNA signatures with hormone profiles, proteomics, and clinical data, aided by machine learning-based decision support systems for easier interpretation and implementation.

While modulating miRNA networks appears to be a promising treatment method for restoring responsiveness in RIF patients, clinical delivery of miRNA mimics or inhibitors (antagomiRs) is still in its early phases. Issues such as target specificity, off-target effects, immunogenicity, and delivery system stability must be addressed [51]. However, the endometrium has a distinct benefit in that it is a highly accessible and hormonally sensitive tissue, making intrauterine administration of miRNA-based treatments a potential option for future study. Furthermore, preclinical models, particularly organoid cultures and endometrial-on-a-chip platforms, should be used to investigate treatment effectiveness and safety in a controlled setting before proceeding to clinical trials.

A last constraint is the incomplete knowledge of ceRNA networks, in which miRNAs interact with long non-coding RNAs (lncRNAs), circular RNAs (circRNAs), and mRNAs in a tissue-specific and time-dependent way. Although recent studies have begun to map these relationships, our understanding of how these networks are disturbed in pathological states such as RIF, endometriosis, and adenomyosis is limited. Future research must use integrative systems biology techniques that include transcriptomics, miRNAomics, epigenomics, and proteomics to build network-level models of endometrial receptivity. Incorporating single-cell RNA sequencing and spatial transcriptomics will also allow for the resolution of miRNA-mediated regulation within different endometrial cell subtypes, improving biomarker specificity and treatment targeting.

Given these constraints, the future of miRNA research in reproductive health must focus on connecting molecular findings to clinical applications via standardized methods, thorough validation, and integrated bioinformatics. Collaboration among academic institutes, fertility clinics, and diagnostic firms is required to build common biobanks, standardized pipelines, and translational trial frameworks. In parallel, public repositories and open-access databases should be enhanced to include proven miRNA–target interactions, polymorphisms, and clinical outcomes linked to endometrial receptivity and IVF success. To summarize, although the present limitations emphasize the complexities of integrating miRNA-based diagnostics and therapies in reproductive health, they also show the molecular class’s transformational promise. With continuing advancements in molecular profiling technology, data analytics, and biomarker validation, miRNAs have the potential to alter the paradigm from empirical IVF techniques to precision-guided, molecularly informed reproductive care.

Artificial intelligence (AI), particularly machine-learning (ML) and deep-learning approaches, is rapidly transforming reproductive care. In the context of endometrial receptivity, AI systems may examine high-dimensional data from transcriptomic, proteomic, and epigenomic profiles to uncover subtle patterns that standard statistical approaches cannot detect. Notably, recent research has created AI-powered miRNA classifiers, such as the PanelChip^®^ platform and MIRA test, that combine circulating miRNA signatures with clinical factors to reliably predict the window of implantation and implantation outcomes. These methods have achieved diagnostic accuracy greater than 90%, highlighting their potential utility in tailoring embryo transfer time and hormone supplementation strategies.

Furthermore, AI-powered integrative frameworks can use multi-omics datasets, endometrial biopsies, uterine fluid, and even embryo culture data to create predictive models for IVF success. The use of neural networks and ensemble-learning techniques is predicted to improve the sensitivity and specificity of endometrial receptivity measurements. As the field advances, AI will most likely offer real-time, non-invasive, and tailored assessments of uterine preparation, paving the path for more targeted and effective ART therapies. Future research should concentrate on external validation, explainable AI models, and regulatory mechanisms to promote clinical translation.

## 2. Materials and Methods

This systematic review was carried out in compliance with the Preferred Reporting Items for Systematic Reviews and Meta-Analyses (PRISMA) 2020 guidelines, and the protocol was prospectively recorded in the PROSPERO database with registration number CRD420251001811. The review aimed to thoroughly assess the role of microRNAs (miRNAs) and other non-coding RNAs (ncRNAs), such as long non-coding RNAs (lncRNAs) and circular RNAs (circRNAs), as molecular regulators and potential biomarkers of endometrial receptivity and RIF.

### 2.1. Search Strategy and Study Selection

A thorough and systematic literature search was carried out to identify eligible studies on the expression, functional relevance, and clinical utility of microRNAs (miRNAs) and other non-coding RNAs (lncRNAs and circRNAs) in relation to endometrial receptivity, implantation biology, and RIF. The search was intended to gather all relevant peer-reviewed publications published between January 2010 and March 2025, including both foundational molecular investigations and recent clinical biomarker research.

Electronic searches were carried out in the following databases: PubMed/MEDLINE, Scopus, and Web of Science, employing a structured search approach based on a combination of Medical Subject Headings (MeSH) and free-text keywords. The search terms included “microRNA”, “miRNA”, “non-coding RNA”, “lncRNA”, or “circRNA”, “endometrial receptivity”, “window of implantation”, “implantation failure”, “recurrent implantation failure”, “RIF”, “biomarker”, “extracellular vesicle”, “liquid biopsy”, “molecular signature”, or “embryo implantation”, and “IVF”, “assisted reproduction”, or “infertility”.

Boolean operators (“AND”/“OR”) and truncation were used to combine search phrases and capture all relevant variations. Search filters were used to limit the results to human studies with full-text availability and publication in peer-reviewed publications. There were no language limits at first, but due to resource constraints, only papers with full texts available in English were included in the final synthesis. To ensure that the search was comprehensive, the reference lists of included publications and pertinent review papers were manually searched for additional suitable studies. In parallel, grey literature sources, such as preprint archives and institutional repositories, were searched for potentially relevant but unpublished or newly accepted research. To ensure methodological integrity, preprints that had not been peer-reviewed at the time of screening were excluded.

All identified citations were entered into Zotero 6.0.37. or deduplication. Two independent reviewers (Reviewer A and Reviewer B) divided the study selection procedure into two sections. In the first phase, titles and abstracts were reviewed to remove unnecessary items. In the second phase, full-text versions of all possibly eligible publications were obtained and compared to the inclusion and exclusion criteria outlined in the review methodology. Disagreements were handled at all stages by discussion or consultation with a third senior reviewer. The entire screening process was documented using a PRISMA 2020 flow diagram, which shows the number of records identified, screened, excluded (with reasons), and eventually included. This approach provides transparency and repeatability in accordance with the best standards for systematic reviews. The PRISMA checklist was used during the study identification and selection procedure.

A total of 28 papers passed the inclusion criteria and were used in the final qualitative synthesis. These studies cover a wide range of study designs (prospective, retrospective, and cross-sectional), biological samples (endometrial tissue, plasma, uterine fluid, saliva, and embryo spent media), molecular methodologies (qRT-PCR, microarray, and RNA sequencing), and clinical phenotypes (receptive versus non-receptive endometrium, IVF success versus RIF, endometriosis-related infertility, etc.).

### 2.2. Eligibility Criteria

This systematic review includes studies that met predefined eligibility criteria and corresponded with the aims outlined in the PROSPERO-registered protocol (CRD420251001811). These criteria were developed to ensure the inclusion of high-quality, relevant material addressing the molecular regulation of endometrial receptivity and implantation outcomes by microRNAs (miRNAs) and other non-coding RNAs (ncRNAs).

To be eligible for inclusion, studies must satisfy the following conditions:

1. Study type: Only original, peer-reviewed research articles were considered. Prospective and retrospective cohort studies, case–control studies, cross-sectional studies, and randomized controlled trials (RCTs) were all considered valid designs. Basic research involving clinical human samples was also considered if the molecular endpoints were well defined. Review articles, editorials, conference abstracts, case reports, commentaries, and animal-only research were not included unless they contained human tissue validation.
2. Population: Studies had to include human participants who were being evaluated or treated for infertility, in vitro fertilization (IVF), embryo transfer, RIF, or conditions known to affect endometrial receptivity. Studies on healthy endometrial controls were also considered if they were compared to non-receptive or infertile populations.
3. Biological samples: Eligible research must disclose molecular analysis of human-derived samples, including the following:
  - Endometrial tissue (biopsies and tissue explants);
  - Uterine fluid: plasma or serum saliva;
  - Embryo wasted its culture medium. These sample types were approved as long as they were collected during relevant times of the menstrual cycle (particularly the mid-secretory or luteal phase) during IVF preparation.
4. Molecular biomarkers: Although miRNAs were the primary focus of the study, studies involving other non-coding RNAs, such as long non-coding RNAs (lncRNAs) and circular RNAs (circRNAs), were also included if they provided functional or correlative evidence relevant to endometrial receptivity, WOI, implantation failure, or embryo–maternal interactions.
5. Analytical methods: The studies had to use validated molecular techniques for RNA profiling and quantification, including but not limited to the following:
  - Quantitative real-time polymerase chain reaction (qRT-PCR);
  - Microarray hybridization;
  - Next-generation sequencing (NGS);
  - Bioinformatic pathway enrichment and target prediction (when combined with experimental validation).
6. Outcomes: Studies needed to report on at least one of the following outcome domains to be included:
  - miRNA or ncRNA expression in receptive and non-receptive endometrium;
  - Diagnostic or predictive performance in terms of implantation outcomes;
  - MiRNAs play a functional role in implantation-related signaling, including HOXA10, LIF-STAT3, and Wnt/β-catenin;
  - Differences in miRNA expression between fertile and infertile populations;
  - Association between miRNAs and IVF success, embryo transfer result, or RIF diagnosis
7. Studies were excluded if they matched any of the following criteria:
  - Non-human animal models were used exclusively, with no equivalent human validation;
  - MiRNAs and other ncRNAs were not assessed, and no molecular data relevant to endometrial function were reported;
  - Concentrated primarily on embryo development or oocyte competency, without looking at the endometrial or implantation environment;
  - Sample-collection timing was unclear or there was no information on the cycle phase.
  - Did not apply a proven molecular quantification approach, and there were no adequate control groups or normalization strategies;
  - Were not published in English or were not available in full-text format.

These qualifying criteria ensured that the studies included in the review were both biologically and clinically relevant, with molecular data that could be meaningfully interpreted in terms of implantation biology and fertility success. To reduce bias and improve methodological integrity, two reviewers applied these criteria consistently and independently.

### 2.3. Data Extraction

Data extraction was carried out methodically and in compliance with the PROSPERO methodology (CRD420251001811) to ensure consistency, transparency, and reproducibility. A standardized data extraction form was created in advance, tested on a selection of eligible trials, and refined to include all relevant methodological, molecular, and clinical characteristics related to endometrial receptivity and implantation outcomes.

Each included study’s data were extracted by two independent reviewers using the finalized extraction form. This method was intended to reduce bias and the possibility of transcription errors. In cases of disagreement between reviewers, the extracted data were cross-checked, debated, and resolved by consensus. When appropriate, corresponding authors were contacted to clarify missing or confusing data points. Each study’s authorship, year of publication, nation or region of study, study type (e.g., prospective, retrospective, and randomized trial), and total sample size were all noted. Population variables, such as inclusion and exclusion criteria, patient diagnoses (e.g., RIF, endometriosis, and unexplained infertility), and control groups (e.g., fertile women or receptive endometrium), were recorded. Where applicable, the menstrual cycle phase or endometrial dating (e.g., mid-secretory phase, LH+7, and progesterone exposure) at the time of sample collection was documented.

The biological source of RNA was determined and classified as endometrial tissue; plasma or serum; uterine fluid; saliva; or embryo culture media. Details about RNA isolation and quantification methods were carefully extracted, such as the molecular platforms used (e.g., qRT-PCR, microarray analysis, and next-generation sequencing), internal or external normalization strategies (e.g., U6 snRNA, SNORD48, and spike-in controls), and whether quality control measures were reported (e.g., RNA integrity assessment and technical replicates). Specific miRNAs or other non-coding RNAs (e.g., lncRNAs and circRNAs) under investigation were identified, along with their expression patterns (upregulated or downregulated) in relation to endometrial receptivity or implantation results. Target genes, signaling pathways, and functional mechanisms (e.g., LIF-STAT3, HOXA10 regulation, and Wnt/β-catenin) were retrieved where accessible. Functional validation (for example, luciferase tests, Western blotting, in vitro knockdown, or overexpression investigations) was also described. To assess the diagnostic or predictive utility of the identified biomarkers, any reported performance metrics were extracted, such as area under the curve (AUC), sensitivity, specificity, positive or negative predictive value, and classification accuracy, particularly in studies that developed diagnostic models or machine learning-based classifiers.

All extracted data were organized into structured summary tables. Table 1, introduced in the Materials and Methods section, summarizes the methodological features of each study, including sample source, molecular method, validation status, and risk of bias categorization. Table 2 presents the main findings of the included studies, organized by biomarker type and clinical context. This organized strategy for data extraction ensured that both the molecular mechanisms and clinical implications of miRNAs and other non-coding RNAs were fully captured and synthesized in accordance with the review’s goals.

### 2.4. Quality Assessment and Risk of Bias

To ensure methodological rigor and reduce the influence of biased evidence, all included studies were subjected to a rigorous assessment of their quality and internal validity. Two independent reviewers used validated tools to assess the methodological soundness of each study, and conflicts were resolved through discussion or arbitration by a third reviewer as needed.

Given the variability in study designs, we used a dual approach for quality assessment. Observational studies, including prospective and retrospective cohorts, as well as case–control studies, were evaluated using a modified Newcastle–Ottawa Scale (NOS) tailored to the molecular biomarker setting. This technique assessed three fundamental domains: group selection, group comparability, and result assessment. Specific criteria included clarity in establishing inclusion and exclusion criteria, recruiting process, explanation of control groups (for example, fertile women or receptive endometrium), adequate sample size, and statistical transparency. For studies that contained diagnostic-accuracy data, such as those testing miRNA-based classifiers, liquid biopsy tools, or predictive biomarkers for endometrial receptivity, the QUADAS-2 (Quality Assessment of Diagnostic Accuracy Studies) tool was also used. This tool focuses on four primary areas: patient selection, index test validity, reference standard, and flow and timing. Each domain was assessed for the possibility of bias and questions about application. The emphasis was on whether the diagnostic model was established and validated in an independent cohort; whether outcome assessors were blinded; and whether performance metrics, including sensitivity, specificity, and area under the curve (AUC), were appropriately reported.

We looked at technical and experimental quality markers in all of the papers we included. These included the use of validated molecular techniques (e.g., qRT-PCR and RNA-seq), RNA quality control procedures, the inclusion of technical replicates, normalization strategies (e.g., endogenous vs. spike-in controls), and the presence of target validation experiments (e.g., luciferase assays and Western blotting). Studies with unclear reporting of these elements were thought to have a higher risk of bias. The preliminary overall risk of bias for each study was classified as low, moderate, or high based on an aggregated assessment of the relevant domains. Table 2 summarizes the appraisal’s findings, providing an overview of methodological characteristics and risk categorization for each study. A majority of the studies were classified as intermediate risk due to constraints such as small sample sizes, a lack of independent validation cohorts, or insufficient methodological reporting. However, numerous recent studies that used multicenter designs, AI-based diagnostic tools, and external validation (e.g., MIRA test and PanelChip) were assessed as low risk and thought to provide higher levels of evidence. No studies were omitted primarily for the danger of bias; nevertheless, quality scores were considered during the interpretation of findings, evidence synthesis, and discussion of translational implications. This methodology enabled a thorough yet critical assessment of the present literature, taking into account both potential developments and methodological limits.

The methodological evaluation of the 28 studies included in this systematic review, as summarized in Table 1, reveals both consistency and significant variability across key design features, reflecting the evolving but maturing landscape of miRNA research in endometrial receptivity and implantation failure. A large proportion of the research used a retrospective observational design, with a smaller but growing number using prospective techniques, notably in more recent publications. Despite increased interest in non-invasive sample techniques, the biological source of RNA varied greatly, with nearly half of the studies investigating endometrial tissue biopsies and the remainder using more accessible fluids, such as plasma, serum, uterine fluid, or saliva. This variation in sample sources presents both potential for non-invasive diagnostics and issues associated with biological comparability.

From a technical standpoint, qRT-PCR was the most common method for RNA quantification, appearing in nearly all included research. This preference reflects the method’s broad availability, high sensitivity, and compatibility with targeted miRNA analysis. However, only a small number of studies supplemented qRT-PCR data with broader transcriptome approaches like RNA sequencing or microarray profiling, limiting the exploratory depth of most studies. In terms of data normalization, most research used endogenous controls, primarily short nuclear RNAs such as U6 or SNORD48. While this technique is extensively employed, it may generate biases due to differences in reference gene expression between tissues and clinical states. Only a few studies used spike-in controls or global mean normalization, which are considered more robust in extracellular RNA analysis, particularly in plasma or exosomal samples.

One significant limitation of the dataset was the lack of independent validation cohorts, which were found in only a few recent studies. This absence reduces confidence in the generalizability of reported biomarker signals and increases the risk of overfitting, especially in studies with small sample sizes. Although multiple studies yielded internally identical results, few undertook external validation in geographically or demographically diverse populations. This methodological gap highlights a key barrier to clinical translation. Most studies were classified as having a moderate risk of bias, owing to sample size limits, single-center recruiting, a lack of blinding during sample processing or outcome assessment, or poor reporting of technical duplicates and quality control methods. Only a small number of studies received a low risk of bias grade, mainly those that used multicenter designs, blinded evaluation processes, or confirmed their findings in independent patient groups. Importantly, no studies were eliminated because of methodological quality; nevertheless, studies assessed as low risk had more interpretive weight in the synthesis of findings and discussion of clinical implications.

Table 1 shows that, while there is a high level of interest in miRNAs and non-coding RNAs as regulators and biomarkers of endometrial receptivity, there is still a pressing need for molecular protocol standardization, improved reporting transparency, and the inclusion of independent validation cohorts. Addressing these methodological shortcomings will be critical in moving miRNA biomarkers from experimental promise to clinical use.

## 3. Results

**Figure 1:**
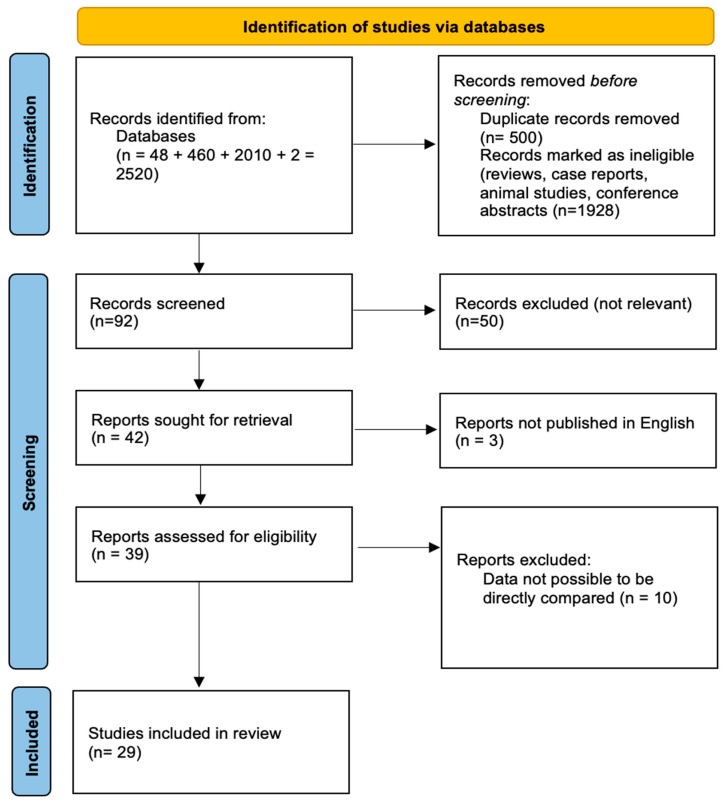
PRISMA flow diagram.

A total of 1264 records were discovered via database searches and other means. After screening and eligibility assessment, 28 studies were chosen for the final qualitative synthesis. The PRISMA 2020 flow diagram (Figure 1) illustrates the study-selection process.

Figure 1 depicts the PRISMA 2020 flow diagram of the study-selection process, including the number of records identified, screened, evaluated for eligibility, and included in the review, as well as the grounds for exclusion.

### Overview of Included Studies

This systematic review comprised 28 papers published between 2011 and 2025, and the papers were selected using the screening and eligibility criteria described in the PRISMA 2020 flow diagram. These studies are part of a rapidly evolving field of research that seeks to understand the regulatory roles and diagnostic potential of microRNAs (miRNAs) and other non-coding RNAs (ncRNAs), such as long non-coding RNAs (lncRNAs) and circular RNAs (circRNAs), in the context of endometrial receptivity, embryo implantation, and RIF. The investigations have a wide geographical scope, with research teams headquartered in Europe, Asia, and North and South America, reflecting global interest in molecular reproductive medicine.

The bulk of the studies used an observational design, with both prospective and retrospective cohorts. These studies were mostly undertaken in clinical populations undergoing in vitro fertilization (IVF) or frozen embryo transfer (FET), frequently comparing groups of patients with successful implantation to those with recurring implantation failure or infertility of unexplained cause. A smaller number of studies used experimental validation, such as functional in vitro assays or gene expression analysis, whereas three studies developed and validated diagnostic models using machine learning or predictive-scoring algorithms. The sample size varied greatly. The majority of studies had less than 50 individuals, highlighting the practical constraints of acquiring timely endometrial biopsies or IVF-associated material. However, a few recent studies have included larger patient populations, particularly those that use plasma or saliva samples for non-invasive biomarker analysis. The total number of patients covered by all included studies surpassed 2000; however, not all offered extensive demographic or clinical classification.

The biological materials studied differed significantly between investigations. Approximately half of the included studies focused on mid-secretory or luteal-phase endometrial biopsies, corresponding to the window of implantation (WOI), which is commonly defined as days LH+6 to LH+9 or progesterone day 5–7 in hormone-replacement cycles. These tissue samples allowed for direct profiling of miRNA expression in the epithelial and stromal compartments. Other investigations used liquid biopsy techniques to extract extracellular RNA from plasma, serum, uterine fluid, saliva, or embryo culture medium. The growing use of non-invasive or minimally invasive sample types represents a larger translational movement toward accessible, reproducible diagnostic techniques for measuring endometrial function.

The majority of research used quantitative real-time PCR (qRT-PCR) to quantify miRNAs, with individual miRNAs being targeted based on previous transcriptome screens or known biological importance. A subset used NGS or microarray systems to conduct broad discovery profiling, which was frequently followed by focused qRT-PCR confirmation. Bioinformatic techniques were commonly used in mechanistic investigations to predict miRNA-mRNA interactions and connect dysregulated miRNAs to implantation-related pathways (e.g., Wnt/β-catenin, HOX signaling, and LIF-STAT3). While the majority of the studies included looked at the difference in miRNA expression between receptive and non-receptive endometrial environments, others focused on functional validation of specific miRNA–gene interactions or clinical associations between circulating miRNA levels and IVF or FET results. Several studies investigated miRNAs as potential diagnostic biomarkers, with performance measures such as AUC, sensitivity, and specificity reported in plasma- and saliva-based trials. However, only a few studies included independent validation cohorts or prospective replication, which has significant implications for the generalizability of their findings.

To consolidate the findings from the included research, a thorough summary table (Table 2) was created. This table summarizes the main parameters, molecular targets, and reported outcomes for endometrial receptivity and implantation failure across the 28 qualifying studies. It is a comprehensive resource for comparing the extent, depth, and findings of miRNA and non-coding RNA research in the context of reproductive implantation.

Table 2 summarizes the expanding body of evidence that non-coding RNAs, particularly microRNAs (miRNAs), play an important role in the regulation of endometrial receptivity and the etiology of RIF. The 28 papers included in this review reveal a consistent pattern: dysregulation of particular miRNAs is closely related with disturbed molecular signaling, changed gene expression profiles, and, ultimately, a non-receptive endometrial phenotype. Many of the most-often-reported miRNAs, including miR-30d, miR-135b, miR-145, miR-29c, and miR-125b, were consistently found to be differentially expressed in receptive and non-receptive endometrial settings. These miRNAs regulate implantation-related pathways such as HOX gene networks, the LIF-STAT3 axis, IGF signaling, Wnt/β-catenin signaling, and ECM remodeling. For example, miR-135b, which is frequently high in women with RIF or endometriosis, directly targets HOXA10, a transcription factor required for stromal cell differentiation and epithelial transition during the window of implantation. Similarly, downregulation of miR-30d, which normally enhances LIF signaling and immunological tolerance, has been linked to molecular receptivity failure in a number of studies.

Importantly, the majority of research went beyond differential expression analysis to give functional evidence for the identified miRNAs’ regulatory activities. Several groups used in vitro models, such as human endometrial stromal cell cultures and trophoblast cell lines, to show that manipulating miRNA levels altered target gene expression and had an impact on key phenotypes like decidualization capacity, adhesion molecule expression, and cytokine secretion. Additional investigations used luciferase reporter assays and Western blotting to validate predicted miRNA–target interactions, adding to the biological significance of these findings. A subgroup of studies investigated the role of long non-coding RNAs (lncRNAs) and circular RNAs (circRNAs) as upstream regulators or miRNA sponges in competitive endogenous RNA (ceRNA) networks. These included substances such as lncRNA H19, NEAT1, and circ_0038383, which were found to alter miRNA activity and thereby influence gene networks involved in endometrial differentiation and immune regulation. Circ_0038383, for example, has been found to increase receptivity by binding to miR-196b-5p and inhibiting the suppression of HOXA9, a gene required for stromal transformation and embryo–endometrial communication. These ceRNA interactions add a new level of complexity and flexibility to gene control during implantation.

The impact of miRNA and ncRNA dysregulation was not only molecular, but also clinically relevant. Most investigations found that altered RNA profiles were associated with implantation failure, low pregnancy rates, or non-receptive histological or transcriptomic profiles. Several studies categorized patients based on IVF outcomes and found that particular miRNAs could identify receptive from non-receptive endometria with good sensitivity and specificity. Plasma-based miRNA classifiers, such as the MIRA test and PanelChip systems, have shown over 90% accuracy in identifying tailored windows of implantation, suggesting clinical value in cycle optimization. Saliva and uterine fluid-derived miRNAs have also shown promise for non-invasive diagnostics, particularly in patients with endometriosis or unexplained infertility. Despite methodological differences in sample source (e.g., endometrial biopsy vs. plasma), molecular platforms (qRT-PCR vs. NGS), and normalization strategies, the convergence of findings across studies supports the existence of common miRNA regulatory signatures governing endometrial function. These characteristics frequently indicate failure in crucial processes such as immunological adaptation, decidualization, hormone signaling, and angiogenesis, all of which are required for a responsive endometrial phenotype. However, the investigation also identifies significant limits. Only a tiny proportion of research used external validation cohorts, and many studies were constrained by small sample numbers, single-center recruitment, or a lack of established techniques for miRNA quantification and normalization. These constraints should be considered when assessing the repeatability and generalizability of reported biomarkers.

In conclusion, the findings described in Table 2 highlight the importance of non-coding RNAs in the molecular landscape of endometrial receptivity. The consistency of identified miRNAs and mechanistic targets across investigations shows biological relevance, as well as translational potential. These findings lay a solid foundation for furthering miRNA-based diagnostics, including cycle personalization and implantation failure risk assessment, while also providing insight into therapeutic targets that could improve IVF outcomes and fertility care in women experiencing unexplained reproductive failure.

A quantitative meta-analysis was not performed since the included papers were highly heterogeneous. Differences in study design, sample size, patient populations, biological sample types and timing, miRNA profiling platforms (qRT-PCR, microarray, and RNA sequencing), normalization procedures, and outcome measures made statistical pooling difficult. Furthermore, differences in the specific miRNAs studied, the definition of endometrial receptivity, and the reporting of diagnostic measures (e.g., AUC, sensitivity, and specificity) added complication. As a result, the findings of this systematic review are provided as a qualitative synthesis, allowing for a thorough and contextualized assessment of molecular pathways, biomarker potential, and clinical relevance across a variety of study contexts.

Diagnostic measures were reported for a subset of the studies included. For example, Cho et al. (2016) found that the miRNA panel had an AUC of 0.79 for distinguishing RIF patients from fertile controls [5]. Similarly, Chen et al. (2023) presented a classifier consisting of seven circulating miRNAs that attained an AUC of 0.89 (95% CI: 0.83–0.95), sensitivity of 82.4%, and specificity of 88.6% in the validation dataset [43]. These measurements emphasize the potential diagnostic value of certain miRNA patterns.

## 4. Discussion

This systematic review summarizes the current evidence on the regulatory roles and diagnostic applications of microRNAs (miRNAs) and other non-coding RNAs (ncRNAs), including long non-coding RNAs (lncRNAs) and circular RNAs (circRNAs), in the context of endometrial receptivity and implantation failure. Twenty-eight studies published between 2011 and 2025 were reviewed, indicating the increased global interest in identifying molecular predictors of successful implantation and possible biomarkers for use in reproductive medicine.

Across investigations, dysregulated miRNA expression was linked to reduced endometrial receptivity and RIF. The most frequently implicated miRNAs, including miR-30d, miR-135b, miR-145, miR-29c, and miR-125b, were shown to regulate important biological pathways governing stromal cell decidualization, hormone responsiveness, immunological regulation, and extracellular matrix remodeling. These miRNAs primarily targeted genes such as HOXA10, LIF, IGFBP1, and MMP26, all of which are required for creating a receptive endometrial environment during the window of implantation (WOI).

Several investigations expanded their analysis to include ceRNA networks, finding that lncRNAs and circRNAs act as competing endogenous regulators, altering miRNA availability and indirectly influencing gene expression. The circ_0038383/miR-196b-5p/HOXA9 axis, as well as the connection between lncRNA H19 and miR-29c, are significant examples of regulatory intricacy in the receptive endometrium, demonstrating a multilayered RNA-based regulation system. In addition to mechanistic discoveries, a subgroup of studies tested miRNAs as non-invasive diagnostic biomarkers and found encouraging findings. Circulating miRNAs in plasma, saliva, and uterine fluid were highly accurate in diagnosing receptive endometrium, RIF risk, and endometriosis-associated infertility. The MIRA test and PanelChip-based classifiers achieved AUCs greater than 0.90 in clinical validation, demonstrating their translational potential.

Despite differences in study design, sample source, and analytical procedures, certain key findings emerged across the literature. Specifically, miRNA-mediated deregulation of conserved implantation pathways appears to be a common trait in non-receptive endometrial conditions, regardless of underlying cause. This convergence lends credence to the resilience of these molecular fingerprints and their prospective use in individualized embryo transfer, cycle optimization, and predictive diagnostics.

### 4.1. Molecular Disruption of Implantation Pathways

Chettiar et al. (2024) [71] conducted a comprehensive transcriptome and pathway analysis in RIF, revealing dysregulation in key signaling cascades, including PI3K-Akt, MAPK, Ras, and cytokine–cytokine receptor connections. These pathways control cell survival, apoptosis, inflammation, and immunological responses, all of which are intricately linked to endometrial receptivity. Dysregulated miRNAs targeting FLT4, IGF2, and BIRC3 have been linked to defective angiogenesis and aberrant endometrial proliferation, supporting the idea that miRNA perturbations disrupt the molecular milieu necessary for embryo implantation. Chen et al. (2024) [46] identified miR-145 and miR-200 family members as suppressors of epithelial–mesenchymal transition (EMT), a crucial change that maintains the endometrium’s morphological plasticity during the window of implantation (WOI). When these miRNAs are dysregulated, they can impede EMT-related pathways, preventing epithelial cells from transitioning into a permissive, receptive phenotype and contributing to implantation failure.

Von Grothusen et al. (2022) [27] extended on this molecular insight by linking miRNA targets to more than 25 KEGG pathways involved in implantation. Upregulated miRNAs in RIF were associated with genes involved in cytoskeletal dynamics and membrane signaling domains (e.g., membrane rafts), whereas downregulated miRNAs influenced kinase networks and chromatin remodeling. The convergence of Wnt, TGF-β, focal adhesion, and apoptosis pathways shows how miRNAs coordinate numerous cellular systems to generate a responsive endometrium. Drissennek et al. (2020) [58] provided more data, demonstrating considerable downregulation of miR-455-3p, let-7b-5p, and miR-424-3p in the receptive phase. These miRNAs are known to influence cell proliferation, migration, and differentiation—processes that must be properly controlled during embryo implantation. The observed downregulation facilitates the transition to a permissive, non-proliferative endometrial state capable of trophoblast invasion. Zhai et al. (2019) [57] brought therapeutic importance to the discussion, demonstrating that metformin, an insulin-sensitizing drug, enhances receptivity in PCOS animals by downregulating miR-135b, a HOXA10 repressor. Because HOXA10 is a master transcriptional regulator of endometrial receptivity, its restoration boosts the expression of downstream targets, including integrins and LIF, which are both required for blastocyst adherence and implantation.

### 4.2. Clinical Relevance of Endometrial miRNA Signatures

Identifying endometrial miRNA profiles with diagnostic and prognostic relevance is a promising step forward in ART customization. MicroRNAs provide a dynamic and less intrusive window into the endometrium’s molecular state during the implantation phase. Their durability in bodily fluids, cell specificity, and regulatory impact on entire gene networks make them excellent candidates for precision diagnostics and treatment stratification.

Xu et al. (2025) [72] presented one of the most compelling clinical uses for molecular diagnostics in ART. Their large-scale retrospective study of over 3600 women found that customized embryo transfer (pET) using Endometrial Receptivity Analysis (ERA) significantly improved clinical pregnancy and live birth rates in both RIF and non-RIF patients. Importantly, patients with a shifted window of implantation (WOI) as determined by transcriptome markers, many of which are controlled by miRNAs, benefited the most from pET. The ERA platform includes genes such as HOXA10, LIF, and IGFBP1, which are known miRNA targets, particularly miR-135b, miR-145, and miR-200a, indicating a close relationship between diagnostic readouts and miRNA-mediated gene regulation.

Opuchlik et al. (2024) [3] stressed the significance of demographics and clinical environment in determining endometrial receptivity. Using gene-expression classifiers to compare women with RIF to controls, they discovered that pre-receptive and early-receptive endometrial states were more common in older women and those with longer infertility durations. These suboptimal profiles were associated with altered expression of important receptivity biomarkers, many of which are known to be miRNA-controlled. For example, miR-181a, miR-29c, and miR-126, which have all been linked to age-related reproductive decline, regulate genes involved in angiogenesis, apoptosis, and inflammatory signaling. This highlights miRNA profiles’ potential as indicators of receptivity, as well as surrogate measures of biological endometrial age and function.

Ruan et al. (2014) and Cheng et al. (2019) [73,74] found that elevated levels of miR-145, miR-126, and miR-200a were substantially associated with non-pregnancy outcomes following ART in women with endometriosis-associated infertility. These miRNAs have been shown to downregulate key genes for decidualization (IGF1R, VEGFA, and HOXA10), which may indicate an underlying molecular resistance to implantation, even in histologically normal endometrium. Their diagnostic profile allows them to stratify endometriosis patients for alternate treatments, such as immune modulation or hormone priming. In terms of diagnostic innovation, Omes et al. (2024) and Juarez-Barber et al. (2023) [47,67] developed non-invasive miRNA-based screening methods by analyzing miRNAs in discarded embryo culture material and peripheral blood. Omes et al. [47] discovered miR-21-5p, miR-372-5p, and miR-373-3p as possible biomarkers for embryo competence and implantation. These miRNAs regulate endometrial receptivity genes, as well as signaling molecules involved in adhesion, invasion, and stromal cell transformation, in addition to embryonic expression. Similarly, Juarez-Barber et al. [67] found that plasma miRNA profiles, which indicate overall reproductive health, were predictive of endometrial status and ART outcomes. These findings suggest liquid biopsy techniques that can eliminate the requirement for invasive endometrial sample while preserving diagnostic accuracy.

Additionally, Chen et al. (2024) and Chettiar et al. (2024) [46,71] created bioinformatic pipelines that combined miRNA profiles with gene expression and protein interaction networks to identify “hub” genes like COL1A2, IGF1R, and FN1. These genes were frequently connected to miR-145, miR-223-3p, and miR-21, which control endometrial stromal remodeling, angiogenesis, and immune surveillance. Such integrated techniques provide multi-omics diagnoses, allowing doctors to categorize patients based on genetic subtypes rather than clinical symptoms alone. Furthermore, Tan et al.’s (2025) [70] findings underlined the involvement of miRNAs in progesterone resistance, a recognized factor relating to implantation failure in frozen embryo transfer (FET) cycles. Elevated levels of miR-497-5p and miR-99b-5p were linked to decreased expression of PGR, FOXO1, and HAND2, which are key regulators of endometrial differentiation under progesterone control. Assessing these miRNA levels before performing FET may help to advise hormone supplementation regimens or timing modifications. Finally, Salmasi et al. (2024) [17] showed that miRNAs not only reflect the current status of endometrial receptivity but also actively shape it via networks including epithelial–mesenchymal transition (EMT), decidualization, immunological tolerance, and vascular adaptability. Their functional categorization of miRNAs, including miR-27a, miR-223-3p, miR-181, and miR-888, provides a mechanistic framework for interpreting miRNA data from tissue and fluid biopsy samples.

### 4.3. The Central Role of HOX Genes and Decidualization Regulation

The transcriptional regulation of endometrial receptivity is orchestrated by a core set of developmental genes, with the homeobox (HOX) gene family, specifically HOXA10, playing a fundamental and non-redundant role [75]. HOXA10 was initially characterized for its role in uterine development and Müllerian duct segmental patterning, but it has since been shown to be a master regulator of adult endometrial function, working downstream of estrogen and progesterone signals [76]. It prepares the endometrium for the blastocyst by regulating stromal differentiation; immunological tolerance; and the production of key adhesion molecules, such as integrin αvβ3, IGFBP-1, and leukemia inhibitory factor (LIF) [77].

Ashary et al. (2020) [18] proposed a comprehensive model for HOXA10’s involvement in implantation, defining its triphasic role as (1) inducing endometrial receptivity in response to ovarian steroid hormones; (2) facilitating decidual transformation in response to embryonic signals; and (3) coordinating trophoblast invasion and placentation via downstream effectors. The cyclical expression of HOXA10 peaks at the mid-secretory phase, which corresponds to the window of implantation. Any departure from this expression pattern, particularly if caused by epigenetic regulation via microRNAs (miRNAs), might significantly impair implantation capability. An increasing number of data link miRNAs to the post-transcriptional silencing of HOXA10, altering this critical sequence of events. Zhai et al. (2019) [57] found that miR-135b binds directly to HOXA10’s 3’ UTR, inhibiting its expression in PCOS models. Metformin therapy restored HOXA10 expression by downregulating miR-135b. This led to increased expression of integrin αvβ3 and IGFBP1, which are hallmarks of a receptive endometrium. This finding emphasizes the therapeutic regulation of the miRNA-HOXA10 relationship as a method for reversing endometrial resistance to implantation.

Multiple investigations in endometriosis-associated infertility—Yang et al. (2018), Wang et al. (2016), and Riyanti et al. (2020) [60,69,78]—showed abnormal expression of HOXA10 in eutopic endometrial tissue despite normal histology. The overexpression of certain miRNAs, such as miR-543, miR-142-5p, miR-146a-5p, and miR-29c, all of which adversely regulate HOXA10, is a common molecular thread running through these studies. Yang et al. [69] discovered that the cyclic fluctuation of miR-543 is disrupted in women with endometriosis, resulting in chronic repression of HOXA10 and poor decidualization. This abnormal expression most likely represents a disordered uterine environment that fails to synchronize with the embryo, resulting in implantation failure.

The ramifications of HOXA10 suppression go beyond the loss of adhesion indicators. Cheng et al. (2019) and Ruan et al. (2014) [73,74] discovered that miR-145 and miR-200a, both overexpressed in infertile endometriosis patients, target not just HOXA10 but also genes involved in angiogenesis (e.g., VEGFA and IGF1R) and stromal remodeling. This multiplex targeting exacerbates the effects of miRNA dysregulation, resulting in a hostile endometrial environment incapable of facilitating embryo implantation or preserving decidual integrity. Menkhorst et al. (2023) [79] revealed that the miRNA miR-19b-3p, while not directly affecting HOXA10, upregulates HOXA9 in trophoblast cells, lowering proliferation and viability. This implies that misregulated stromal-derived miRNAs might alter trophoblast function in a paracrine way, emphasizing the relevance of maternal–fetal interaction mediated by HOX-controlled pathways.

Furthermore, Fu et al. (2024) used single-cell RNA sequencing to identify subpopulations of stromal cells in thin vs. normal endometrium with altered HOX gene expression profiles, including HOXA10 and HOXA11, as well as dysregulated TNF and MAPK signaling. These data demonstrate that HOX gene regulation is not uniform across the endometrium, but rather geographically and temporally heterogeneous, and its disruption is associated with decreased cellular preparation for decidual transformation [80]. Epigenetic and endocrine signaling also alter the expression of HOXA10. Tan et al. (2025) [70] found that miR-497-5p and miR-99b-5p, which are implicated in progesterone resistance, target the progesterone receptor and downstream effectors such as HOXA10. Because HOXA10 expression is progesterone-dependent, its reduction by these miRNAs reinforces the progesterone-resistant phenotype, which is frequently found in frozen embryo-transfer failures or in patients with repeated implantation failure. Finally, Azarpoor et al. (2022) and Chettiar et al. (2024) [65,71] describe regulatory networks involving lncRNAs and circRNAs, which add another degree of complexity. In these models, lncRNAs like H19 and SMIM25 operate as competitive endogenous RNAs (ceRNAs), sequestering miRNAs such as miR-138, miR-200c, and miR-424-5p, and thereby indirectly restoring HOXA10 expression and other implantation-critical genes. Disruption of these ceRNA networks increases the availability of repressive miRNAs, continuing the silencing of HOXA10 and slowing down the decidualization process.

### 4.4. Systems Biology and Single-Cell Insights

While bulk transcriptome investigations have greatly aided our understanding of endometrial receptivity, they necessarily obscure the cellular heterogeneity and spatial complexities of the endometrial landscape. The endometrium, a complex, temporally dynamic tissue made up of epithelial, stromal, immunological, and vascular cells, relies on coordinated cellular interactions to facilitate implantation. Recent advancements in systems biology and single-cell RNA sequencing (scRNA-seq) have opened up a new arena in reproductive research, allowing for high-resolution mapping of endometrial cell types, states, and gene regulatory networks, including those governed by miRNAs and noncoding RNAs.

Fu et al. (2024) [80] used single-cell transcriptomics to investigate the molecular differences between thin endometrium (TE-RIF) and normal endometrium (NE-RIF) in women suffering from recurrent implantation failure. They identified unique cellular phenotypes and transcriptome patterns in epithelial, stromal, and immune cells. Notably, the TE-RIF group revealed abnormal activation of the TNF, MAPK, and apoptotic pathways, whereas the NE-RIF group showed disruption in oxidative phosphorylation and lipid metabolism, despite having morphologically normal thickness. These system abnormalities were supported by differential expression of critical regulatory genes, many of which are known or expected miRNA targets, including miR-21, miR-30a, and miR-223, which are all involved in inflammation, epithelial remodeling, and metabolic adaptation. What separates single-cell techniques, such as those used by Fu et al., is their ability to resolve cell-type-specific regulatory processes, particularly those related to decidualization and immune–endometrial interaction. For example, subsets of stromal cells in TE-RIF patients showed reduced expression of decidual markers (PRL and IGFBP1) and higher stress-response signatures, indicating insufficient decidual transformation. In parallel, immune cell clusters, particularly uterine natural killer (uNK) cells and macrophages, exhibited altered chemokine profiles and cytokine signaling, most likely altering the immunological-tolerant milieu required for blastocyst adoption.

To supplement these biological findings, systems biology studies, such as those by Chettiar et al. (2024) and Chen et al. (2024), [46,71] used gene regulatory network reconstruction and protein–protein interaction (PPI) modeling to uncover the hierarchical architecture of miRNA–target connections in RIF. Chettiar et al. found that miRNAs such as miR-21-5p, miR-145, and miR-223-3p regulate critical nodes in the PI3K-Akt, MAPK, and TGF-β signaling pathways. Their network analysis found that these miRNAs control not only individual genes but also entire gene clusters involved in angiogenesis (VEGFA and FLT1), immunological regulation (CXCL12 and IL10), and apoptosis (BCL2 and CASP9).

These findings were replicated in the ceRNA networks published by Azarpoor et al. (2022) and Li et al. (2022), [64,66] who built lncRNA-miRNA-mRNA interaction networks to investigate how non-coding RNAs can indirectly affect gene expression by acting as miRNA sponges. For example, lncRNAs such as H19, NEAT1, and SMIM25 have been demonstrated to sequester restrictive miRNAs (e.g., miR-424-5p, miR-138, and miR-29c), thereby increasing the expression of downstream genes required for differentiation and immunological tolerance. When these ceRNA networks are disrupted in RIF patients, miRNAs that repress receptivity genes become more bioavailable, perpetuating abnormal molecular features.

Furthermore, von Grothusen et al. (2022) [27] used pathway enrichment to examine miRNA-expression datasets and discovered a pattern of miRNA dysregulation across more than 25 KEGG pathways. Their system-level approach revealed that miRNAs influence numerous aspects of endometrial function, including cellular adhesion (by integrins), signal transduction (via Wnt and MAPK), and tissue remodeling (via MMPs and ECM proteins). This wide range of targets highlights miRNAs’ enhanced regulatory capacity, since a single miRNA can affect dozens of genes and pathways, especially when paired with changed lncRNA and circRNA expression levels. Furthermore, integrating these multi-omics datasets opens up the possibility of developing receptivity classifiers using machine-learning techniques. Juarez-Barber et al. (2023) [67] found that miRNA expression profiles, when paired with clinical factors and transcriptomic signatures, can predict implantation success with accuracies greater than 90%. These classifiers, which were trained on system-level data, show potential for non-invasive diagnostics, embryo selection, and personalized therapy.

### 4.5. Angiogenesis, Immune Crosstalk, and ceRNA Networks

A receptive endometrium is distinguished by its ability to change its vasculature and immunological landscape in preparation for implantation. This intricate transition necessitates closely controlled interplay among stromal, epithelial, endothelial, and immunological cells. MicroRNAs (miRNAs) and long non-coding RNAs (lncRNAs) are key components of this regulatory apparatus, acting as post-transcriptional and epigenetic modulators of gene expression. Recent discoveries show that these small non-coding RNAs control not only specific gene targets, but also global regulatory networks via ceRNA (competing endogenous RNA) interactions, resulting in a multilayered system that governs angiogenesis and immune tolerance during the window of implantation (WOI). Successful implantation is dependent on the creation of a responsive vascular niche, which is defined by enhanced permeability, regulated vasodilation, and the formation of new capillaries to promote embryonic invasion and nutrient supply. This process is primarily driven by vascular endothelial growth factor A (VEGFA) and its receptors, which are directly regulated by multiple miRNAs.

Cheng et al. (2019) and Ruan et al. (2014), [73,74] discovered upregulation of miR-126, miR-145, and miR-200a in the endometria of women with endometriosis-related infertility. These miRNAs target and repress VEGFA, IGF1R, and PI3K/Akt signaling components, resulting in poor angiogenesis, decreased stromal cell proliferation, and faulty decidual transition. Their increased expression correlates with lower conception rates and demonstrates how aberrant miRNA expression leads to an anti-angiogenic uterine environment, even in histologically normal endometrium. He et al. (2024) [68] underscored the molecular signature of deficient angiogenesis in RIF patients, correlating downregulation of angiogenesis-promoting miRNAs to poor endometrial perfusion and immunological dysfunction. They discovered that miRNAs such as miR-30a, miR-132, and miR-145 were variably expressed in decidualizing stromal cells, leading to altered production of angiocrine signals and decreased endothelial cell migration.

The maternal immune system must be functionally reprogrammed in order to facilitate semi-allogeneic embryo implantation without triggering a rejection response. This includes the recruitment of uterine natural killer (uNK) cells, regulatory T cells (Tregs), and macrophages, all of which contribute to immunomodulation and tissue remodeling. miRNAs influence these processes via controlling the expression of cytokines, chemokines, HLA molecules, and immunological checkpoints. Salmasi et al. (2024) grouped miRNAs into functional immunological categories. MiR-223-3p, miR-888, and miR-376a regulate cytokine networks, antigen presentation, and macrophage polarization. Examples include IL-10 and TGF-β. These miRNAs affect not only immune cells, but also endometrial epithelial and stromal expression of adhesion molecules such as ICAM-1, VCAM-1, and CD274 (PD-L1), promoting immunological tolerance during early implantation [17].

Tan et al. (2025) [70] provided a hormonal viewpoint by associating progesterone resistance, a well-known barrier to immunological tolerance and decidualization, with miR-497-5p and miR-99b-5p expression. These miRNAs reduced critical progesterone action mediators such as PGR, FOXO1, and HOXA10, resulting in inflammatory skewing and poor stromal differentiation. Fu et al. (2024) [80] supported this by demonstrating, using single-cell RNA sequencing, that immune–endometrial interaction is substantially disturbed in patients with thin endometrium. Reduced expression of chemokines such as CXCL12 and CCL21, both of which are regulated by miRNAs, was associated with lower immune cell infiltration and tolerance mechanisms. These molecular abnormalities were cell-type specific, highlighting the importance of local regulatory control at the maternal–fetal interface.

The significance of ceRNA networks in reproductive molecular biology is quickly evolving, with lncRNAs and circRNAs acting as molecular sponges, binding miRNAs and blocking them from suppressing mRNA targets. This indirect gene regulation enhances the post-transcriptional control provided by miRNAs and complicates the functional genome. Azarpoor et al. (2022), [64] created a comprehensive ceRNA network in RIF patients, revealing that downregulation of key lncRNAs, such as NEAT1, SMIM25, and H19, resulted in an increase in bioavailable miRNAs, including miR-29c, miR-146a, and miR-200c, which are known to suppress decidualization, angiogenesis, and immune tolerance. This dysregulation changed the endometrial gene expression profile away from a responsive phenotype. Notably, NEAT1 has been found to influence IL-8 production and macrophage polarization, suggesting a direct relationship between the ceRNA network and immunological preparedness.

Chettiar et al. (2024), [71] expanded on this by finding essential nodes in PPI networks influenced by miRNA-lncRNA interactions, such as FLT1, BIRC3, and TIMP3—genes important for vascular integrity, apoptotic control, and extracellular matrix remodeling. Their systems biology approach illustrated how perturbations in the ceRNA network cause many receptivity-related pathways to collapse at the same time. Li et al. (2022), [66] modified the ceRNA network model to incorporate circRNAs, identifying circ_0002375 and circ_0002233 as putative regulators of miR-200a and miR-146a, respectively. These circRNAs were dramatically downregulated in women with RIF, limiting their ability to buffer the inhibitory effect of these miRNAs on HOXA10, LIF, and IGFBP1, contributing to implantation failure.

### 4.6. Non-Invasive miRNA Biomarkers and Diagnostic Potential

The incorporation of molecular biology into clinical reproductive medicine has shifted toward non-invasive assessment of endometrial receptivity, with the goal of overcoming the constraints of traditional histological staging and endometrial biopsies. MicroRNAs (miRNAs), due to their stability in body fluids, tissue-specific expression, and strong regulatory control over implantation-related pathways, have emerged as promising diagnostic indicators in ART. A trend toward miRNA analysis in uterine fluid, serum/plasma, and spent embryo culture media is changing the landscape of customized embryo transfer (pET) and real-time endometrial readiness monitoring. Drissennek et al. (2020) and Grasso et al. (2020), [58,59] pioneered the use of uterine-fluid miRNA profiling to identify miRNAs that represent the molecular status of the endometrial epithelium and stroma during the WOI. They discovered that miR-30d, let-7b, and miR-424 were considerably different between receptive and non-receptive endometria, which corresponded to the expression of important adhesion molecules and cytokines. These miRNAs are known to influence EMT, stromal decidualization, and pro-inflammatory responses, and their presence in uterine secretions provides a dynamic and non-invasive indicator of endometrial health.

Omes et al. (2024) [47] made significant contributions to this field by profiling miRNAs in spent embryo culture media, which represent both embryonic competence and maternal–embryonic communication. Their discovery of miR-21-5p, miR-372-5p, and miR-373-3p as indicators of implantation success emphasizes the importance of bidirectional transmission between the embryo and the endometrium. These miRNAs, produced by viable blastocysts, may interact with endometrial receptors or modify local immunological and vascular responses, resulting in a favorable implantation environment. The capacity to detect embryo viability and mother receptivity using culture media is a key step toward integrated embryo–endometrium diagnostics.

Juarez-Barber et al. (2023), [67] extended the field of miRNA-based diagnostics to peripheral blood, proposing a liquid biopsy technique to assess endometrial receptivity and ART prognosis. Their investigation discovered circulating miRNAs implicated in EMT, cell adhesion, and immunoregulation that were associated with implantation results. This strategy is consistent with accumulating evidence that systemic reproductive markers, including inflammation-modulating miRNAs (e.g., miR-146a, miR-21, and miR-223), reflect uterine receptivity and may serve as pre-transfer biomarkers for cycle optimization [67]. Chen et al. (2024) and Chettiar et al. (2024), [46,71] offered an additional biological explanation for these tactics by finding hub genes, such as COL1A2, IGF1R, and FN1, that are controlled by miRNAs found in both endometrial tissue and external fluids. These miRNAs are entrenched in pathways that control matrix remodeling, stromal invasion, and decidual immune tolerance, making them excellent endometrial status indicators. Notably, miR-145, miR-223-3p, and miR-21 were identified as key regulators with diagnostic and therapeutic implications.

The combination of miRNA data with transcriptomic, proteomic, and clinical characteristics enables the building of highly accurate predictive models. Machine-learning classifiers trained on miRNA expression profiles, such as those investigated by Juarez-Barber et al., may accurately predict implantation success with high sensitivity and specificity. These models allow patients to be classified as responsive or non-receptive, which informs decisions about embryo transfer time, embryo selection, and adjuvant therapy. Furthermore, studies such as Salmasi et al. (2024) and Li et al. (2022) [17,66] have emphasized the functional categorization of miRNAs into biological modules: angiogenesis (miR-126 and miR-132), decidualization (miR-181 and miR-154), immune tolerance (miR-888 and miR-223-3p), and epithelial remodeling. Mapping patient-specific miRNA expression against these functional axes enables mechanism-based diagnostics, which extends beyond simple biomarker panels to individualized endometrial phenotyping.

Several papers in this review reported diagnostic performance indicators for miRNAs or miRNA panels. For example, Cho et al. (2016) [5] found that a polymorphism-based miRNA risk model had a sensitivity of 76.3% and a specificity of 72.6%. Similarly, Chen et al. (2023) [43] created a prediction model based on seven serum miRNAs that had an AUC of 0.89, showing strong discriminative capacity. These findings indicate miRNAs’ potential use not just as pathophysiological indicators but also as non-invasive diagnostic tools in therapeutic settings.

In addition to the biomaterials examined in the studies, such as endometrial tissue, uterine fluid, plasma, and extracellular vesicles, current research has recommended menstrual blood as a new, non-invasive source of biomarkers for measuring endometrial receptivity. Although it cannot be gathered within the implantation window, obtaining it at the start of the embryo transfer cycle may offer useful prognostic data. However, there have been no human studies that precisely examine miRNA expression patterns in menstrual blood in connection to implantation results. Future research in this area may provide intriguing new routes for early cycle-specific receptivity evaluation.

### 4.7. Clinical Implications

The integration of molecular diagnostics, particularly those focusing on microRNAs (miRNAs) and non-coding RNAs, is reshaping the clinical approach to infertility and embryo-implantation failure. The information presented in this systematic analysis highlights a paradigm change from simply morphological or hormonal assessments of the endometrium to a molecularly driven, customized model of endometrial receptivity. These findings have numerous clinically relevant implications for both diagnostic and therapeutic approaches in assisted reproductive technology (ART). First, identifying miRNA signatures associated with the window of implantation (WOI) provides a framework for more precise embryo transfer timing. Traditional calendar-based or morphological staging frequently fails to account for molecular misalignment of the endometrium and embryo. Xu et al. (2025) and Opuchlik et al. (2024) [3,72] found that up to 25–30% of women may have a displaced WOI, particularly those with RIF or who are of advanced maternal age. Incorporating miRNA profiling into ERA-like systems or as an add-on may enable doctors to detect receptive states more accurately and guide the use of customized embryo transfer (pET), thereby reducing unsuccessful cycles and increasing live birth rates.

Second, miRNAs’ significant role in regulating progesterone responsiveness, stromal differentiation, and immunological tolerance proposes new diagnostics and therapeutic targets for disorders such as progesterone resistance, endometriosis-associated infertility, and PCOS-related receptivity abnormalities. Elevated levels of miR-135b, miR-145, or miR-543, as identified in multiple investigations, may serve as molecular markers for endometrial resistance, indicating the need for hormone priming, anti-inflammatory pre-treatment, or tailored endocrine support prior to embryo transfer.

Third, the rising role of non-invasive liquid biopsy procedures that use miRNAs produced from blood or embryo culture media is a viable alternative to invasive endometrial biopsies. These approaches, which have been confirmed by studies such as Omes et al. (2024) and Juarez-Barber et al. (2023), [47,67] allow doctors to conduct real-time functional assessments of endometrial readiness and embryo–endometrium synchrony without disturbing the cycle. This advancement is especially essential for patients undergoing frozen embryo transfer (FET), which requires precise synchronization, as well as those who are at risk of endometrial damage. Furthermore, the incorporation of ceRNA network insights (as demonstrated by Azarpoor et al. (2022) and Chettiar et al. (2024), [64,71] paves the way for therapeutic manipulation of lncRNAs and circRNAs that mitigate the impacts of harmful miRNAs. Such techniques could include using miRNA mimics, inhibitors, or lncRNA-based therapies to restore receptivity in resistant endometrial profiles. This precision technique shows potential in treating tough cases, including patients with chronic endometritis, thin endometrium, and unexplained RIF.

Clinically, our data support stratifying patients based on genetic endometrial phenotypes rather than just morphological or endocrine variables. For example, a patient with normal endometrial thickness but increased miR-126 and miR-145 may be diagnosed with an anti-angiogenic, non-receptive endometrium, necessitating a different treatment strategy than a thin endometrium with normal miRNA levels. This stratification improves customization and cycle efficiency, and it may alleviate the emotional and financial difficulties associated with several ART failures. Finally, new systems biology and single-cell research have recommended the development of multi-omics, AI-driven receptivity prediction models, which could someday be used in reproductive clinics to enhance decision-making. These models could use patient-specific miRNA expression, hormone profiles, and endometrial imaging to dynamically guide treatment planning, representing a breakthrough in precision reproductive medicine.

In conclusion, translating molecular findings into clinical practice offers a significant potential to move beyond the “one-size-fits-all” approach in ART. Clinicians can identify, predict, and treat implantation failure more effectively by leveraging the regulatory complexity of miRNAs and ceRNA networks. As the field progresses toward precision care, molecular receptivity profiling has the potential to be a game-changer in improving implantation outcomes and producing healthy pregnancies in even the most difficult infertility situations.

## 5. Strengths and Limitations

This systematic review provides a thorough synthesis of the most recent and high-impact research on the function of microRNAs (miRNAs), long non-coding RNAs (lncRNAs), and competitive endogenous RNA (ceRNA) networks in endometrial receptivity and implantation outcomes. This review’s merits include molecular depth, breadth of source integration, and clinical translational significance. One of the primary strengths is the diverse range of studies presented. This study reflects the complexity of molecular regulation underlying endometrial receptivity by combining data from in vitro functional assays, in vivo animal models, single-cell transcriptomics, bulk RNA-seq, and bioinformatics network studies. Furthermore, the inclusion of studies that assessed both endometrial tissue and non-invasive biological fluids (e.g., serum, uterine fluid, and wasted embryo culture media) broadens the diagnostic implications and emphasizes their clinical utility.

Another key strength is the emphasis on mechanistic and clinical viewpoints. The review explores how miRNAs modulate key signaling pathways, such as PI3K-Akt, MAPK, TGF-β, and Wnt. It also examines how these molecular insights translate into clinical practice in RIF, personalized embryo transfer (pET), endometriosis, and progesterone resistance. The inclusion of recent, high-quality papers from 2020 to 2025 guarantees that the review represents the most recent scientific breakthroughs, including the use of cutting-edge technologies like single-cell RNA sequencing (scRNA-seq) and integrated ceRNA modeling. Furthermore, by explicitly referencing specific miRNA-lncRNA-mRNA axes and their downstream phenotypic consequences (e.g., decidualization, angiogenesis, and immune modulation), the review provides researchers and clinicians with detailed molecular targets for potential diagnostic or therapeutic investigation.

However, several limits must be recognized. To begin with, the diversity of study design and technique across the collected literature makes standard interpretation difficult. Variations in miRNA detection technologies (qPCR vs. microarrays vs. next-generation sequencing), sample types (tissue vs. fluid), cycle phase, and patient groups (age and cause of infertility) may create bias and impede cross-comparison. Second, while our review identified a number of intriguing miRNA biomarkers, quantitative synthesis (i.e., meta-analysis) was not possible due to variable outcome measurements and a lack of standardized effect sizes across studies. As a result, while the review contains valuable qualitative insights, it is unable to quantitatively confirm the prediction potential of individual miRNAs for clinical outcomes such as implantation or pregnancy rates.

Another restriction is that many of the molecular pathways addressed are still in the preclinical or early translation stages. Several studies have proven the regulatory effects of miRNAs on target genes in cell lines or murine models, but few have validated these findings in large-scale human clinical trials. This limits the immediate clinical application and emphasizes the need for additional translational research. Furthermore, the functional redundancy and pleiotropy of miRNAs hamper attempts to assign causality to individual molecules. Many miRNAs regulate many pathways at the same time, and even tiny changes in expression levels might have context-dependent consequences depending on cellular state, hormonal environment, or embryo quality. Finally, while our evaluation used a thorough search approach and included all relevant full-text articles up to March 2025, it is likely that unpublished data, non-English publications, or grey literature offering useful insights were unintentionally excluded.

## 6. Conclusions

This systematic review emphasizes the vital function of microRNAs (miRNAs) and associated non-coding RNA networks in the molecular regulation of endometrial receptivity, which is essential for successful embryo implantation and assisted reproductive technology (ART) outcomes. MiRNAs have emerged as significant regulators of critical implantation-related processes, including decidualization, angiogenesis, immunological regulation, epithelial–mesenchymal transition, and hormone responsiveness. The review reveals that an abnormal expression of miRNAs such as miR-145, miR-135b, miR-126, miR-543, and miR-223-3p is consistently associated with impaired endometrial receptivity in clinical contexts such as RIF, endometriosis-related infertility, and progesterone resistance. These chemical changes disturb the expression of essential receptivity genes such HOXA10, LIF, IGF1R, and VEGFA, affecting the delicate immune–vascular balance required for embryo–maternal communication.

The integration of ceRNA networks, which include long non-coding RNAs (lncRNAs) and circular RNAs (circRNAs), introduces a new level of complexity and therapeutic potential. These interactions affect miRNA bioavailability and gene expression, impacting pathways such as TGF-β, PI3K-Akt, MAPK, and Wnt signaling. Equally noteworthy is the findings’ translational potential. The discovery of non-invasive miRNA biomarkers in uterine fluid, serum, and embryo culture media offers up new possibilities for molecular diagnostics, allowing for real-time and individualized assessment of endometrial receptivity without the need for invasive biopsies. These techniques have the potential to alter the clinical management of infertility by improving embryo transfer timing, enhancing embryo selection, and guiding tailored therapy options.

Despite methodological heterogeneity and the preliminary nature of certain findings, the aggregate evidence highlights the promise of miRNA-based diagnostics and therapies as essential components of precision reproductive care. Future research should look at verifying these biomarkers in large, prospective clinical cohorts and investigating targeted interventions to modify miRNA activity for therapeutic benefit. To summarize, miRNAs are not only molecular gatekeepers of implantation, but also useful instruments for improving ART success. Their incorporation into clinical protocols has the potential to revolutionize the standard of care for women suffering from unexplained infertility, RIF, or endometrial dysfunction, ushering in a new age of genuinely customized reproductive healthcare.

## Figures and Tables

**Table 1:** Methodological characteristics and quality assessment of the included studies.

Author, Year	Study Design	Sample Size	Sample Source	Molecular Method	Normalization Strategy	Validation Cohort	Risk of Bias
**Revel et al., 2011 [52]**	Retrospective	11 RIF-IVF patients vs. 5 fertile women	Serum/saliva/plasma	qRT-PCR	U6/SNORD/endogenous control	Not reported	Moderate
**Chen et al., 2016 [53]**	Prospective	Human: 16 women with elevated P vs. 9 with normal P; Mouse: groups treated with miR-125b agomir vs. NC/water	Serum/saliva/plasma	qRT-PCR	U6/SNORD/endogenous control	Not reported	Moderate
**Cho et al., 2016 [5]**	Retrospective	120 RIF patients vs. 234 fertile controls	Endometrial tissue	qRT-PCR	U6/SNORD/endogenous control	Not reported	Moderate
**Liu et al., 2017 [54]**	Prospective	6 RIF patients vs. 6 fertile controls (3 each for microarray; 3 each for qRT-PCR validation)	Serum/saliva/plasma	qRT-PCR	U6/SNORD/endogenous control	Not reported	Moderate
**Di Pietro et al., 2018 [55]**	Prospective	15 CE patients vs. 15 healthy controls (serum and endometrial samples)	Serum/saliva/plasma	qRT-PCR	U6/SNORD/endogenous control	Not reported	Moderate
**Xu et al., 2019 [56]**	Prospective	RNA-Seq: 5 RIF patients vs. 5 controls Validation: 14 RIF patients vs. 17 controls	Endometrial tissue	qRT-PCR	U6/SNORD/endogenous control	Not reported	Moderate
**Zhai et al., 2019 [57]**	RCT	60 PCOS patients with metformin vs. 60 PCOS patients without metformin	Serum/saliva/plasma	qRT-PCR	U6/SNORD/endogenous control	Not reported	Moderate
**Drissennek et al., 2020 [58]**	Retrospective	Discovery cohort: 5 non-receptive vs. 15 receptive patients Validation cohort: 27 non-receptive vs. 25 receptive patients (total 123 RIF patients analyzed)	Serum/saliva/plasma	qRT-PCR	U6/SNORD/endogenous control	Not reported	Moderate
**Grasso et al., 2020 [59]**	Prospective	58 ELB samples from 40 healthy ovum donors (each in natural and HRT cycles)	Serum/saliva/plasma	qRT-PCR	U6/SNORD/endogenous control	Not reported	Moderate
**Riyanti et al., 2020 [60]**	Prospective	14 infertile women vs. 9 fertile controls	Endometrial tissue	qRT-PCR	U6/SNORD/endogenous control	Not reported	Moderate
**Li et al., 2021 [61]**	Prospective	Group A: 22 fertile women (LH+2 vs. LH+7) Group B: 51 IVF patients (hCG+2 vs. hCG+7; 18 conceived, 33 not)	Serum/saliva/plasma	qRT-PCR	U6/SNORD/endogenous control	Not reported	Moderate
**Zhao et al., 2021, aging [62]**	Prospective	18 RIF patients vs. 16 fertile controls (validation cohort) GEO datasets: circRNA (8 vs. 8), miRNA (7 vs. 5), mRNA (43 vs. 42)	Endometrial tissue	qRT-PCR	U6/SNORD/endogenous control	Not reported	Moderate
**Zhao et al., 2021 (Reprod. Biol. Endocrinol.) [63]**	Prospective	20 RIF patients vs. 10 fertile controls	Serum/saliva/plasma	qRT-PCR	U6/SNORD/endogenous control	Not reported	Moderate
**Azarpoor et al., 2022 [64]**	Prospective	10 RIF+PBMCs vs. 10 RIF vs. 10 fertile patients	Endometrial tissue	qRT-PCR	U6/SNORD/endogenous control	Not reported	Moderate
**Babian et al., 2022 [65]**	Prospective	30 pregnant vs. 30 non-pregnant RIF patients (PRP-treated)	Serum/saliva/plasma	qRT-PCR	U6/SNORD/endogenous control	Not reported	Moderate
**Li et al., 2022 [66]**	Retrospective	1143 FET cycles (Group A: 290 HT only; Group B-D: 293 + 310 + 250 = 853 with GnRHa pretreatment)	Serum/saliva/plasma	qRT-PCR	U6/SNORD/endogenous control	Not reported	Moderate
**Von Grothusen et al., 2022 [27]**	Prospective	33 RIF patients vs. 15 fertile controls	Endometrial tissue	qRT-PCR	U6/SNORD/endogenous control	Not reported	Moderate
**Chen et al., 2023 [43]**	Retrospective	111 in model training; 73 in validation	Serum/saliva/plasma	qRT-PCR	U6/SNORD/endogenous control	Not reported	Moderate
**Dabi et al., 2023 [7]**	Prospective	153 women with endometriosis (79 infertile, 74 fertile) vs. 31 healthy controls	Serum/saliva/plasma	qRT-PCR	U6/SNORD/endogenous control	Not reported	Moderate
**Juarez-Barber et al., 2023 [67]**	Prospective	4 adenomyosis patients vs. 4 fertile controls	Serum/saliva/plasma	qRT-PCR	U6/SNORD/endogenous control	Not reported	Moderate
**Omes et al., 2023 [47]**	Prospective	53 embryos from 16 IVF patients (BLOK vs. NE/DG; 5 pools each group)	Serum/saliva/plasma	qRT-PCR	U6/SNORD/endogenous control	Not reported	Moderate
**Soczewski et al., 2023 [6]**	Retrospective	n = 20 (10 RPL vs. 10 RIF patients)	Serum/saliva/plasma	qRT-PCR	U6/SNORD/endogenous control	Not reported	Moderate
**Alset et al., 2024 [12]**	Prospective	65 total (30 FGR patients vs. 35 healthy controls)	Serum/saliva/plasma	qRT-PCR	U6/SNORD/endogenous control	Not reported	Moderate
**Bendifallah et al., 2024 [8]**	Prospective	153 total (16 SPE vs. 137 non-SPE endometriosis phenotypes)	Serum/saliva/plasma	qRT-PCR	U6/SNORD/endogenous control	Not reported	Moderate
**Chen et al., 2024 [46]**	Retrospective	200 total: 150 successful implantations vs. 50 failed implantations	Serum/saliva/plasma	qRT-PCR	U6/SNORD/endogenous control	Not reported	Moderate
**He et al., 2024 [68]**	Prospective	54 women (FET with clinical pregnancy); human and mouse models used	Serum/saliva/plasma	qRT-PCR	U6/SNORD/endogenous control	Not reported	Moderate
**Yang et al., 2024 [69]**	Retrospective	22 IVF patients (no separate control group; all underwent mock FET cycles)	Serum/saliva/plasma	qRT-PCR	U6/SNORD/endogenous control	Not reported	Moderate
**Tan et al., 2025 [70]**	Retrospective	80 total (40 RIF patients vs. 40 controls)	Endometrial tissue	qRT-PCR	U6/SNORD/endogenous control	Not reported	Moderate

This table highlights the methodological characteristics of the 28 studies included in the systematic review of microRNAs and other non-coding RNAs in endometrial receptivity and implantation failure. It describes the study design (prospective or retrospective), sample size and clinical grouping, biological source of RNA (e.g., endometrial tissue, plasma, and saliva), molecular technique used for RNA quantification (e.g., qRT-PCR), normalization strategies used, presence of an independent validation cohort, and overall assessment of risk of bias. The risk of bias was evaluated using a modified Newcastle–Ottawa Scale (NOS) and classified as low, moderate, or high. The majority of studies used qRT-PCR as their principal analytical platform, with normalization using endogenous controls such U6 or SNORD RNAs. A majority of studies were classed as intermediate risk due to sample size limits, single-center designs, or a lack of validation.

**Table 2:** Characteristics, molecular targets, and functional outcomes of included studies investigating non-coding RNAs in endometrial receptivity and implantation failure.

Author, Year	Type of Study	Sample Size	Inclusion Criteria	Exclusion Criteria	Type of microRNA	Target Gene/Protein	Receptivity
**Revel et al., 2011 [52]**	retrospective	11 RIF-IVF patients vs. 5 fertile women	Women < 41 years, ≥4 IVF-ET failures, good ovarian reserve (FSH < 8 mIU/mL), >8 oocytes per retrieval, normal uterine cavity, endometrial thickness ≥ 8 mm	Infertility from severe male factor, oral contraceptives or IUD use, breastfeeding, hormonal therapy during study cycle	13 miRNAs (↑ miR-23b, miR-145, miR-99a, miR-27b, miR-652, miR-139-5p, miR-195, miR-342-3p, miR-150, miR-374b; ↓ miR-32, miR-628-5p, miR-874)	miR-145 → N-cadherin, H2AFX, netrin-4; miR-23b → sFRP-4	Impaired
**Chen et al., 2016 [53]**	prospective	Human: 16 women with elevated P vs. 9 with normal P; Mouse: groups treated with miR-125b agomir vs. NC/water	Women aged 23–38 years, regular cycles, undergoing IVF-ET for male factor infertility, no uterine pathology	PCOS, endometriosis, ovarian tumor, hydrosalpinx, steroid use within 3 months	miR-125b	MMP26	Impaired
**Cho et al., 2016 [5]**	retrospective	120 RIF patients vs. 234 fertile controls	Women with RIF: failure after ≥2 fresh IVF-ET cycles, >10 cleaved embryos, high-quality embryos; <40 years old; Korean ethnicity	Anatomical/chromosomal/hormonal/infectious/autoimmune/thrombotic causes of implantation failure; Müllerian anomaly, hypothyroidism, chromosomal abnormality, antiphospholipid syndrome; male partners with abnormal semen/karyotype/hormones	miR-146aC>G, miR-149C>T, miR-196a2T>C, miR-499A>G	CCND2 (Cyclin D2), COL8A2, NF2, FZD4 (via miR-146a-3p alleles)	Impaired
**Liu et al., 2017 [54]**	Prospective	6 RIF patients vs. 6 fertile controls (3 each for microarray; 3 each for qRT-PCR validation)	Women with ≥3 IVF-ET failures with high-grade embryos transferred; fertile women with ≥1 live birth for controls	N/A	circRNA sponges: hsa_circRNA_070616, _103716, etc. → miR-574-5p	miR-574-5p (indirect target: MACC-1, others via sponge activity)	Impaired
**Di Pietro et al., 2018 [55]**	Prospective	15 CE patients vs. 15 healthy controls (serum and endometrial samples)	Infertile women undergoing hysteroscopy with confirmed histological CE (study group); healthy women (controls)	N/A	miR-27a-3p, miR-124-3p	IGF1 (miR-27a-3p), IL11 (miR-124-3p)	Impaired
**Xu et al., 2019 [56]**	Prospective	RNA-Seq: 5 RIF patients vs. 5 controls Validation: 14 RIF patients vs. 17 controls	Women aged 25–35 years RIF: ≥3 embryo transfers with ≥4 good-quality embryos Controls: peritubal infertility, pregnant within 1–2 ETs	uterine anomaly, impaired glucose tolerance, abnormal thyroid function, antiphospholipid antibodies, or other diseases	86 differentially expressed miRNAs (e.g., miR-424-5p, miR-488-3p, miR-548o-3p, miR-30d-5p, miR-124-5p, etc.)	Multiple targets via ceRNA network (e.g., DOCK8, MYH11, IL6 pathways; miRNAs target via lncRNAs like NEAT1, TRG-AS1, SMIM25, H19, etc.)	Impaired
**Zhai et al., 2019 [57]**	RCT	60 PCOS patients with metformin vs. 60 PCOS patients without metformin	Women diagnosed with PCOS (Rotterdam criteria), undergoing IVF/ICSI, with insulin resistance	N/A	miR-491-3p, miR-1910-3p (↓ with metformin)	HOXA10, ITGB3	Improved
**Drissennek et al., 2020 [58]**	Retrospective	Discovery cohort: 5 non-receptive vs. 15 receptive patients Validation cohort: 27 non-receptive vs. 25 receptive patients (total 123 RIF patients analyzed)	Women with ≥3 failed embryo transfers (mean 9.1 failed attempts; 9.8 non-implanted embryos), undergoing hormone replacement and endometrial biopsy in implantation window	N/A	miR-455-3p, miR-4423-3p (↓ in receptive); miR-152-3p, miR-155-5p (↑ in implantation failure)	miR-155-5p → TGFβ signaling, leukocyte extravasation; miR-152-3p → cell migration and adhesion genes	Impaired
**Grasso et al., 2020 [59]**	Prospective	58 ELB samples from 40 healthy ovum donors (each in natural and HRT cycles)	Healthy women (18–34 years), regular cycles, normal BMI (19–29), normal karyotype, participation in ovum donation program	N/A	Receptivity-associated ↑: miR-30d-5p, miR-873-3p, miR-345-5p, miR-30d-3p, miR-141-3p, miR-30b-3p, miR-223-3p, miR-582-5p; ↓: various others in non-receptive phases	MAPK8, FOXO1, HOXA11, ITGB3, RASSF2, IGF2, KIF11, ATP5B, VCAM1, MMP2, MMP9, SLC1A1, SLC7A1 (differ by cycle and miRNA)	Improved
**Riyanti et al., 2020 [60]**	Prospective	14 infertile women vs. 9 fertile controls	Infertile: women undergoing IVF with normal hormonal profiles, sperm analysis, mid-secretory phase endometrium (≥8 mm), regular cycles, normal uterus imaging. Fertile: ≥1 normal pregnancy/delivery, attending for Pap smear/screening	N/A	miR-135b	HOXA10	Impaired
**Li et al., 2021 [61]**	Prospective	Group A: 22 fertile women (LH+2 vs. LH+7) Group B: 51 IVF patients (hCG+2 vs. hCG+7; 18 conceived, 33 not)	Age < 40, regular menstrual cycles, normal uterine cavity, no history of infertility (Group A); IVF patients on first/second cycle, no uterine abnormalities (Group B)	IVF patients with no embryo transfer or missing data excluded	12 conserved EV-sncRNAs: 11 miRNAs (e.g., miR-503-5p, miR-196a-5p, and miR-18a-5p) and 1 piRNA (piR-hsa-456); hsa-miR-362-3p ↑ in non-pregnant IVF patients	TGF-β pathway, Hippo signaling, immune response, ECM, cell junction pathways	Improved (in fertile and pregnant cycles); Impaired (↑ miR-362-3p in non-pregnant)
**Zhao et al., 2021 (Aging) [62]**	Prospective	18 RIF patients vs. 16 fertile controls (validation cohort) GEO datasets: circRNA (8 vs. 8), miRNA (7 vs. 5), mRNA (43 vs. 42)	Women undergoing IVF-ET RIF: ≥3 IVF-ET failures Controls: clinical pregnancy after 1–2 ETs for tubal infertility	endometrial disease, hydrosalpinx, PCOS; all with normal cycles, hormone levels, endometrial thickness	miR-196b-5p, miR-424-5p (↓)	HOXA9 (via miR-196b-5p), PBX1, HOXA3 (via miR-424-5p)	Impaired
**Zhao et al., 2021 (Reprod Biol Endocrinol) [63]**	Prospective	20 RIF patients vs. 10 fertile controls	Women aged 25–40; regular menstrual cycles (25–35 days); BMI 18.5–24.9; normal hormone profile (FSH, LH, and E2); infertility due to male or tubal factors	Endometrial/uterine pathology (fibroids, adenomyosis, endometritis, etc.); PCOS; hydrosalpinx; abnormal karyotypes; autoimmunity; endocrine disorders; recent contraceptive use or IUD (within 6 months)	miR-30d-5p	SOCS1 (↑ in RIF), LIF, p-STAT3 (↓ in RIF)	Impaired
**Azarpoor et al., 2022 [64]**	Prospective	10 RIF+PBMCs vs. 10 RIF patients vs. 10 fertile patients	30–35 y, ≥2 RIF, normal endometrium in controls	Tubal/male/unexplained infertility, recurrent miscarriage, infections, systemic/uterine pathologies	miR-199a-5p, miR-125b-5p	LIF, FGFR-2	Improved
**Babian et al., 2022 [65]**	Prospective	30 pregnant vs. 30 non-pregnant RIF patients (PRP-treated)	Women with ≥3 IVF failures and ≥1–2 high-quality embryos per transfer; frozen–thawed embryo transfer cycles; PRP treatment	Anatomical, chromosomal, and hormonal causes of RIF (e.g., hyperprolactinemia, luteal insufficiency, and thyroid disease); systemic diseases; infections	miR-21-3p (↑ in pregnant), miR-21-5p, miR-494-3p, miR-145-5p	RBPMS → SMAD4 inhibition; CCAR1 → reduced apoptosis/proliferation regulation	Improved
**Li et al., 2022 [66]**	Retrospective	1143 FET cycles (Group A: 290 HT only; Group B-D: 293 + 310 + 250 = 853 with GnRHa pretreatment)	Women aged 18–40 with regular cycles (26–35 days), ovulatory, undergoing HRT-FET cycles	Contraindications to estrogen/progesterone, intrauterine adhesions, congenital uterine anomalies, donor oocyte use	miR-124-3p	IL-6, IL-11	Improved
**Von Grothusen et al., 2022 [27]**	Prospective	33 RIF patients vs. 15 fertile controls	Women aged 18–42; RIF: ≥3 failed IVF/ICSI cycles with high-quality embryos Fertile: ≥1 spontaneous pregnancy, regular cycles (21–35 days), no hormonal contraception for ≥3 months prior to sampling	Systemic, endocrinological, or gynecological diseases; infertility; ongoing pregnancy; breastfeeding; >2 miscarriages; regular NSAID use	61 dysregulated miRNAs in UF: Downregulated: miR-486-5p, miR-92b-3p (validated) Upregulated: miR-320a, miR-127-3p, miR-224-5p, etc.	VEGFA, CDH1 (E-cadherin), PI3K/AKT, JAK/STAT, TGF-β, Wnt, estrogen signaling pathways (predicted targets)	Impaired
**Chen et al., 2023 [43]**	Retrospective	111 in model training; 73 in validation	Women aged 21–45; BMI >18.5; ≥1 good-quality frozen blastocyst; no ovulatory disorders, endometriosis, myomas, polyps, or hydrosalpinx	N/A	let-7g-5p, let-7b-5p, miR-423-5p, miR-122-5p, miR-143-3p, miR-375-3p, miR-191-5p	IGF2R (via let-7g-5p), CCND2 (via miR-143-3p), cell cycle pathways	Improved
**Dabi et al., 2023 [7]**	Prospective	153 women with endometriosis (79 infertile; 74 fertile) vs. 31 healthy controls	Women with pelvic pain suggestive of endometriosis; diagnosis confirmed by MRI or laparoscopy; fertile or infertile status assessed	Prior surgery for endometriosis, unclear fertility status, male factor infertility	34 dysregulated salivary miRNAs (e.g., miR-6818-5p, miR-498, miR-1910-3p, miR-3119, miR-501-5p, etc.)	Predicted: PI3K/Akt, JAK/STAT, EGFR, MAPK, Wnt/β-catenin signaling pathways	Impaired
**Juarez-Barber et al., 2023 [67]**	Prospective	4 adenomyosis patients vs. 4 fertile controls	Women aged 18–45; secretory endometrial biopsies; validated adenomyosis diagnosis; fertile controls from oocyte donation program	No hormonal treatments within 3 months; absence of other gynecological pathology	Secretory EV-miRNAs: miR-21-5p, miR-24-3p, miR-26a-5p, miR-92a-3p, miR-92b-3p, miR-200c-3p, miR-423a-5p Gestational EV-miRNAs: miR-21-5p, miR-26a-5p, miR-30a-5p, miR-30c-5p, miR-222-3p, miR-423a-5p	PTEN, PLAGL2, MDM4, CELF1 (downregulated in adenomyosis organoids)	Impaired
**Omes et al., 2023 [47]**	Prospective	53 embryos from 16 IVF patients (BLOK vs. NE/DG; 5 pools each group)	Women undergoing IVF (mean age 37.3 ± 3.4 y); embryos cultured up until day 5/6; standard grading per Istanbul Consensus	N/A	hsa-miR-661 hsa-miR-21–5p hsa-miR-372–5p	hsa-miR-661: associated with epithelial morphogenesis, apoptosis (MDM2/p53 pathway), cell structure integrity; potential role in impairing implantation	Impaired
**Soczewski et al., 2023 [6]**	Retrospective	n = 20 (10 RPL vs. 10 RIF patients)	Age 28–40, regular cycles (28–31 days), no infectious, endocrine or anatomic disease	Infectious, endocrine, anatomic disease	miR-17-5p miR-21-5p miR-193b-3p	miR-17-5p → TXNIP miR-21-5p → TXNIP, IL-1β miR-193b-3p → NLRP3	Impaired in RPL group; Improved in RIF group
**Alset et al., 2024 [12]**	Prospective	65 total (30 FGR vs. 35 healthy controls)	Pregnant women aged 19–39, recruited 2018–2021, FGR diagnosed by Doppler ultrasound	IVF, fetal chromosomal/congenital anomalies, previous pregnancies	miR-125a (rs12976445) miR-365b (rs121224) miR-33a (rs9620000) miR-149 (rs2292832)	miR-33a → β-catenin, GSK3 miR-125a → WNT2 miR-365b → PPP5C, Dvl2, VGLL4 miR-149 → WNT3a, TNKS, WNT1	Impaired
**Bendifallah et al., 2024 [8]**	Prospective	153 total (16 SPE vs. 137 non-SPE endometriosis phenotypes)	Women aged 18–43 with pelvic pain suggestive of endometriosis; confirmed endometriosis by laparoscopy and/or MRI	Suspected adenomyosis on MRI	89 miRNAs total signature; Key miRNAs: miR-4421, miR-3153, miR-3974, miR-4632-3p, miR-4674, miR-6511a-5p, miR-190a-5p	PI3K/Akt, Wnt/β-catenin, VEGF, MAPK, NF-κB pathways	Impaired
**Chen et al., 2024 [46]**	Retrospective	200 total: 150 successful implantations vs. 50 failed implantations	Infertile women undergoing IVF with HRT cycles and personalized embryo transfer; endometrial biopsy performed	N/A	143 miRNAs used to build classifiers (e.g., miR-145, miR-155-5p, miR-20b-5p, and miR-718)	IGF1R, ZEB1/2, genes involved in embryo–endometrium crosstalk, preeclampsia, and tissue remodeling	Improved
**He et al., 2024 [68]**	Prospective	54 women (FET with clinical pregnancy); human and mouse models used	Women aged 20–39; natural cycle FET; BMI 18–25; infertility due to tubal/male factors; regular cycles	Hormonal treatment within 3 months; endometriosis, adenomyosis, hydrosalpinx, endometrial lesions; endometrial thickness < 7 mm on hCG day	miR-135a-5p	HOXA10, BMPR2(also influenced by CEBPD)	Impaired
**Yang et al., 2024 [69]**	Retrospective	22 IVF patients (no separate control group; all underwent mock FET cycles)	Women undergoing hormone-replacement therapy (HRT) FET; assessed at 120 ± 4 h post-progesterone	N/A	~100 miRNAs analyzed via MIRA; specific miRNAs include miR-30b, miR-181, miR-223-3p, miR-21, miR-22	Not gene-specific; platform evaluates miRNAs reflecting receptivity states and WOI displacement	Impaired in all initial cycles; Improved after personalized progesterone adjustment in 91% (20/22)
**Tan et al., 2025 [70]**	Retrospective	80 total (40 RIF patients vs. 40 controls)	Women <40 y/o undergoing IVF-ET; ≥3 failed transfers with high-quality embryos (RIF group); vs. pregnancy in one transfer (control)	Chromosomal abnormalities, genital malformations, mental illness, endometritis, polyps, PCOS, thyroid/pituitary dysfunction, major organ diseases, immune disorders, hepatitis, malignancy	miR-31	N/A	Impaired

This table highlights the key methodological characteristics and biological findings from the 28 studies included in this systematic review. The table for each study includes the year of publication, study design (e.g., retrospective and prospective), participant grouping and sample size, inclusion and exclusion criteria, the class and identity of microRNAs or non-coding RNAs studied, validated or predicted gene targets, and the reported functional impact on endometrial receptivity. Receptivity was defined as compromised in studies when differential expression of the ncRNA was linked with molecular or clinical indications of implantation failure, poor endometrial differentiation, or altered signaling cascades (e.g., HOXA10, LIF-STAT3, ECM remodeling, and immunological modulation).
